# Silver Sulfobenzoate Coordination Polymers as Bioactive Dopants in Antibacterial and Antibiofilm Cellulose Films

**DOI:** 10.1021/acsami.6c00401

**Published:** 2026-06-04

**Authors:** Rafaela G. Cabral, Tiago A. Fernandes, Chris H. J. Franco, Paula Jorge, Ivo M. F. Bragança, Ana Catarina Sousa, Nuno Cerca, Alexander M. Kirillov

**Affiliations:** † MINDlab: Molecular Design & Innovation Laboratory, Centro de Química Estrutural, Institute of Molecular Sciences, Instituto Superior Técnico, 72971 Universidade de Lisboa, Av. Rovisco Pais, Lisboa 1049-001, Portugal; ‡ Departamento de Engenharia Química, ISEL − Instituto Superior de Engenharia de Lisboa, Instituto Politécnico de Lisboa, R. Conselheiro Emídio Navarro, 1, Lisboa 1959-007, Portugal; § Departamento de Ciências e Tecnologia (DCeT), Universidade Aberta, Lisboa 1000-013, Portugal; ∥ Institute of Low Temperature and Structure Research, Polish Academy of Sciences, Okólna 2, Wroclaw 50-422, Poland; ⊥ Centre of Biological Engineering, 56059 University of Minho, Campus de Gualtar, Braga 4710-057, Portugal; # LABBELS−Associate Laboratory, University of Minho, Braga/Guimarães 4710-057, Portugal; ∇ IDMEC, Instituto Superior Técnico, Universidade de Lisboa, Av. Rovisco Pais, Lisboa 1049-001, Portugal; ○ CIMOSM, ISEL − Instituto Superior de Engenharia de Lisboa, Instituto Politécnico de Lisboa, R. Conselheiro Emídio Navarro, 1, Lisboa 1959-007, Portugal

**Keywords:** molecular design, functional materials, coordination polymers, antimicrobial biopolymers, bacterial biofilms

## Abstract

Antimicrobial resistance constitutes a critical global health issue, underscoring the need for novel materials designed to inhibit bacterial proliferation and biofilm growth. The present study focuses on the development of antibacterial and antibiofilm biopolymer materials based on bioactive silver­(I) coordination polymers (bioCPs). Two new bioCPs, [Ag­(msba)]_n_ (**CP1**) and [Ag­(Ksba)­(H_2_O)_2_·2H_2_O]_n_ (**CP2**), were assembled from silver­(I) precursors and two structurally analogous sulfobenzoate linkers, namely 4-(methylsulfonyl)­benzoic acid (Hmsba, C_8_H_8_O_4_S) or 4-sulfobenzoic acid monopotassium salt (KHsba, C_7_H_5_O_5_K). To create hybrid biopolymer formulations with different rates of degradation and silver release, these two compounds were used as bioactive dopants in thin films based on ethylcellulose [EC]_n_ or cellulose acetate [CA]_n_. Biopolymer films doped with **CP1** and **CP2** demonstrated a remarkable antimicrobial activity against Gram-positive (*S. aureus* and *S. epidermidis*) and Gram-negative (*E. coli* and *P. aeruginosa*) bacteria. The results were particularly promising for the inhibition of bacterial adhesion and biofilm formation, especially for biopolymers doped with **CP2**, with reductions up to 3.5 log CFU/cm^2^ (99.97% reduction in biofilm growth). By describing new bioCPs and derived biopolymer films made from renewable polysaccharides, this study expands the use of coordination polymers as components of antibacterial and antibiofilm hybrid materials.

## Graphical abstract

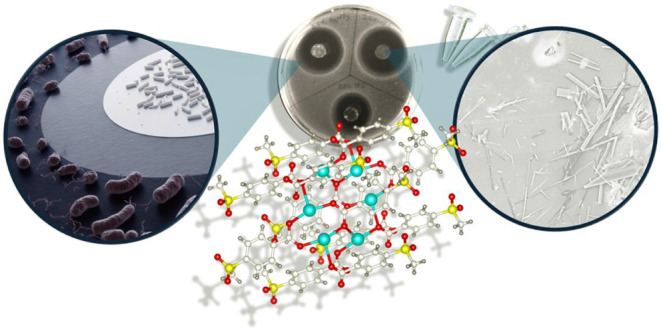

## 1 Introduction

Bacterial biofilms are complex microbial communities that attach to surfaces or each other and are protected by an extracellular matrix. This matrix functions as a shield, defending bacteria from antimicrobials and the immune system.^1–4^ Biofilms not only protect bacteria from environmental stressors but also promote antibiotic resistance and improve nutrition availability and communication, enabling them to survive in different environments.^5–7^ Commonly present in water, soil, and all surface types, biofilms are the major causes of infections in humans and animals. Hence, biofilms present significant challenges in healthcare and industrial applications, underscoring the need for novel materials designed to inhibit bacterial proliferation and biofilm growth.^1,7,8^

Bioactive coordination polymers (bioCPs) represent emerging examples of such antibacterial materials,^9–14^ which are composed of biocidal metal cores and/or bioactive organic linkers.^15–17^ Notably, silver stands out among other biocidal metals since it has strong inhibitory effects on a wide spectrum of microorganisms, including both Gram-positive and Gram-negative bacteria.^18–24^ Silver is especially remarkable because it neutralizes bacteria without damaging mammalian cells and exhibits relatively low toxicity to humans.^25,26^ Different formulations, including Ag^+^ ions, nanoparticles, and silver sulfadiazine, are commonly used as topical silver-based antimicrobials.^27–29^

While the exact mechanism of antibacterial action is still not totally clear, the release of silver­(I) plays a crucial role. Silver­(I) can pass through cell membranes, disrupting their function and inhibiting bacterial growth.^9,30^ The use of ligands that can strongly bind to active silver­(I) ions can be beneficial for improving the performance of the Ag-based antimicrobial systems. These ligands stabilize the silver species within the matrix, slowing its release while prolonging the antimicrobial efficacy. The degradation behavior of hybrid biopolymer materials that are doped with silver compounds is closely linked to how these compounds are released in a controlled way via diffusion, matrix degradation, and/or swelling mechanisms.^31–34^ These processes often work together to create a long-lasting release profile that can inhibit the bacteria from growing around the hybrid material by creating a protective barrier and an inhibition zone.^9,15^ Consequently, this can prevent bacterial accumulation and biofilm formation. In such circumstances, biobased polymers and derived hybrid materials represent an attractive class of antibacterials.^35,36^

For example, cellulose derivatives are excellent candidates for hybrid biopolymer films because of their abundance from different biomass sources, low cost, good stability, and biodegradability.^37,38^ In particular, ethylcellulose (EC) and cellulose acetate (CA) feature linear polymer structures and hydrophilic functional groups and can be easily cross-linked. Other characteristics of these biopolymers include biocompatibility, film-forming ability, mechanical strength, and chemical stability with a controllable permeability. This combination of features facilitates the use of EC and CA for the preparation of hybrid biopolymer materials with incorporated antibacterial agents and relevance in the biomedical and food packaging industries.^39–41^

Following our interest in preparing novel functional biomaterials and combining both the synthetic and antimicrobial approaches, the current work describes the synthesis of new silver­(I) bioCPs, the preparation of hybrid bioCP-doped biopolymer films, as well as their antibacterial and antibiofilm features. Hence, two new bioCPs, formulated as [Ag­(msba)]_n_ (**CP1**) and [Ag­(Ksba)­(H_2_O)_2_·2H_2_O]_n_ (**CP2**), were assembled from 4-(methylsulfonyl)­benzoic acid (Hmsba, C_8_H_8_O_4_S) or 4-sulfobenzoic acid monopotassium salt (KHsba, C_7_H_5_O_5_K) as sulfobenzoate linkers, and incorporated as bioactive dopants into hybrid biopolymer films based on [EC]_n_ or [CA]_n_. The selection of sulfobenzoate linkers as primary building blocks was governed by the following reasons: (i) commercial availability and low cost, (ii) presence of −COOH and −SO_3_K/SO_2_CH_3_ groups that provide several different sites for potential coordination and act as hydrogen bond donors, (iii) sulfonyl/sulfonate groups can enhance aqueous solubility of the resulting coordination compounds, and (iv) recognized antibacterial activity of sulfobenzoic acid derivatives. Hence, by reporting new bioCPs and hybrid biopolymer films, this study integrates key aspects of coordination and materials chemistry, while expanding the potential applications of coordination polymers as effective antimicrobial agents.

## 2 Results and Discussion

### 2.1 Preparation of BioCPs

The compounds [Ag­(msba)]_n_ (**CP1**) and [Ag­(Ksba)­(H_2_O)_2_·2H_2_O]_n_ (**CP2**) were self-assembled from an acetonitrile–water reaction mixture composed of silver­(I) nitrate, 4-(methylsulfonyl)­benzoic acid (Hmsba, C_8_H_8_O_4_S) or 4-sulfobenzoic acid monopotassium salt (KHsba, C_7_H_5_O_5_K), and aqueous ammonia (Scheme 1). The molecular formulas and structures of the resulting compounds were established based on elemental analysis, FTIR spectroscopy (Figures S1–S4, SI), single-crystal and powder X-ray diffraction (PXRD, Figure S5). See SI for structural details.

**Scheme 1:**
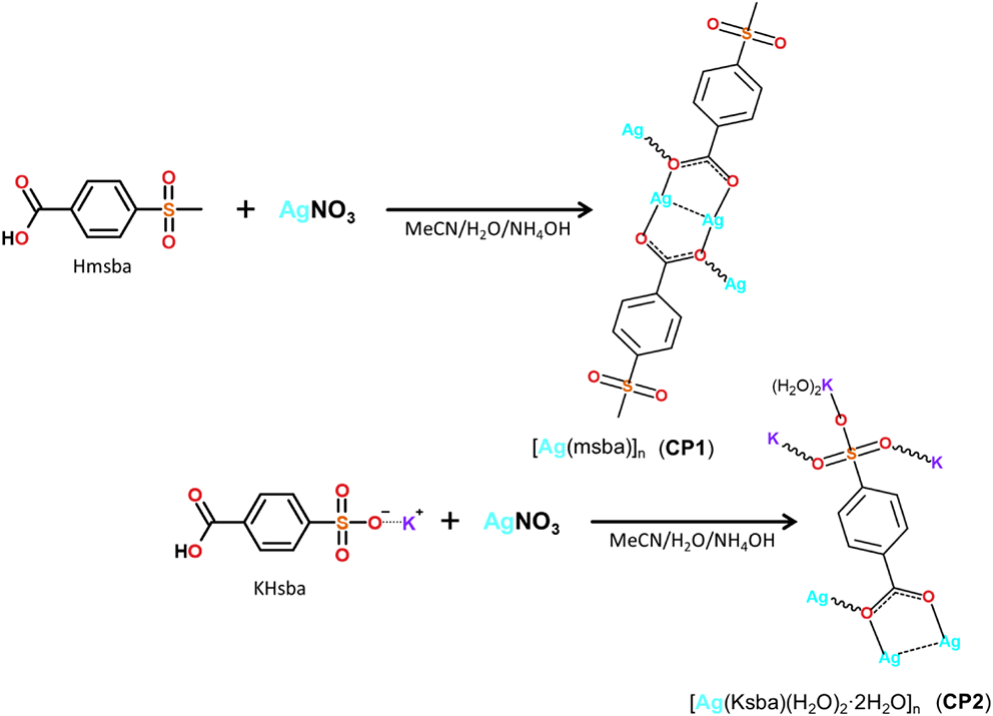
Synthesis of Compounds **CP1** and **CP2**

### 2.2 Preparation of Biopolymer Films

Two types of hybrid biopolymer films were produced by adding bioCPs as dopants into the biopolymer precursors (EC or CA with PEG), thereafter undergoing polymerization in Petri dishes (Scheme 2). Our previously described protocols were adapted.^9,15^ In brief, biopolymer films were prepared using mixtures of CA:acetone:PEG and EC:ethanol:PEG at a ratio of 1:10:0.01 (m/v/m). The polymer precursors were dissolved in the respective solvent under stirring at 200 rpm and 60 °C in a closed flask. After full homogenization, PEG was added, and the mixtures were stirred for an additional 15 min. Low concentrations (1, 2.5, and 5 wt %) of bioCP dopants, previously mechanically ground into powders, were incorporated into the solutions, ultrasonically treated for 5 min, and, subsequently, stirred at 300 rpm at 60 °C for 15 min to achieve maximum homogenization. The obtained solutions were then poured into Petri dishes (0.1 m diameter) and allowed to dry by casting at 30 °C. After 24 h of drying, final samples of biopolymer films, designated as **CP1** ^(1%)^@[EC]_n_, **CP1** ^(2.5%)^@[EC]_n_, **CP1** ^(5%)^@[EC]_n_, **CP2** ^(1%)^@[EC]_n_, **CP2** ^(2.5%)^@[EC]_n_, and **CP2** ^(5%)^@[EC]_n_ (EC series), along with **CP1** ^(1%)^@[CA]_n_, **CP1** ^(2.5%)^@[CA]_n_, **CP1** ^(5%)^@[CA]_n_, **CP2** ^(1%)^@[CA]_n_, **CP2** ^(2.5%)^@[CA]_n_, and **CP2** ^(5%)^@[CA]_n_, (CA series) (Figure 1), were obtained, removed from the Petri dishes, and kept at room temperature in a desiccator. The biopolymer film samples were usually of ∼1 mm thickness. For comparative purposes, the related negative control samples (undoped [EC]_n_ and [CA]_n_) and the positive control samples (AgNO_3_^(1%)^@[EC]_n_, AgNO_3_^(2.5%)^@[EC]_n_, AgNO_3_^(5%)^@[EC]_n_, AgNO_3_^(1%)^@[CA]_n_, AgNO_3_^(2.5%)^@[CA]_n_, AgNO_3_^(5%)^@[CA]_n_, Hmsba^(1%)^@[EC]_n_, Hmsba^(2.5%)^@[EC]_n_, Hmsba^(5%)^@[EC]_n_, Hmsba^(1%)^@[CA]_n_, Hmsba^(2.5%)^@[CA]_n_, Hmsba^(5%)^@[CA]_n_, Hsba^(1%)^@[EC]_n_, Hsba^(2.5%)^@[EC]_n_, Hsba^(5%)^@[EC]_n_, Hsba^(1%)^@[CA]_n_, Hsba^(2.5%)^@[CA]_n_, Hsba^(5%)^@[CA]_n_) were also produced. Before analyses, all the biopolymer films were stored in a desiccator.

**Scheme 2:**
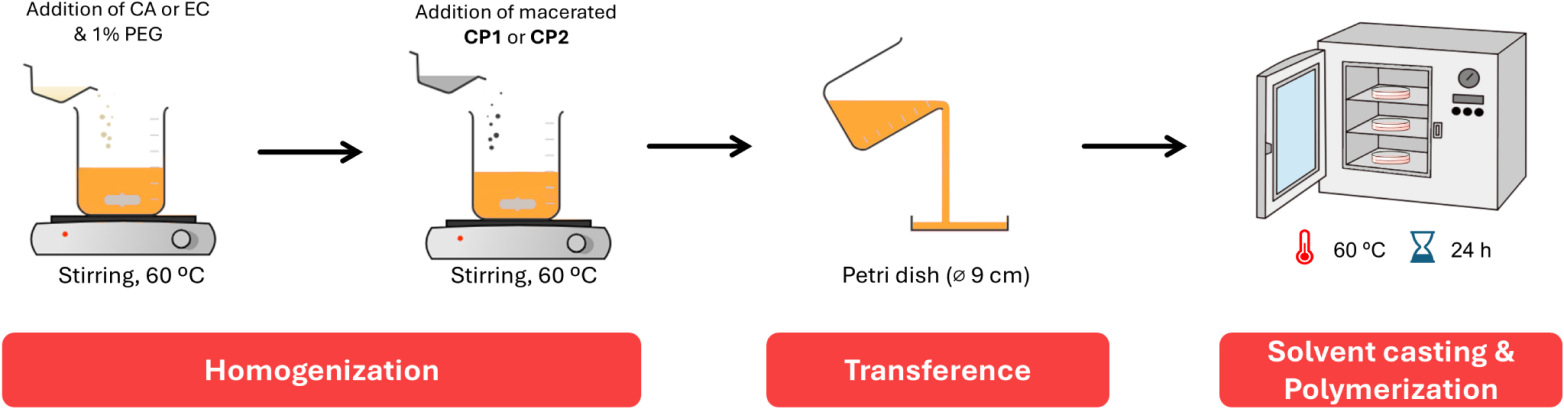
Preparation of BioCP-Doped Biopolymer Films

**Figure 1:**
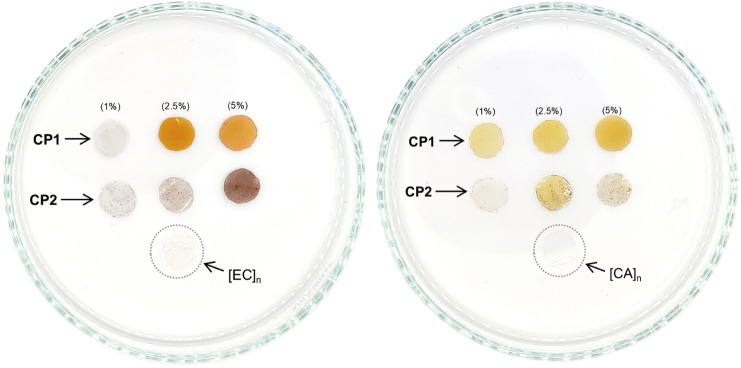
Photographs of the as-prepared coupons of [EC]_n_ (left) and [CA]_n_ (right) biopolymer films doped with **CP1** and **CP2** (1, 2, and 5%).

The produced biopolymer films were characterized by Fourier transform infrared spectroscopy coupled with attenuated total reflectance (FTIR-ATR, Figures S1–S4, SI), thermogravimetric analysis (TGA, Figures S6–S9, SI), and scanning electron microscopy with energy-dispersive X-ray analysis (SEM-EDX). The latter showed a predominantly homogeneous distribution of bioCPs throughout the films; however, some samples exhibited localized regions containing crystalline agglomerates. In addition to silver ion release tests, water absorption and polymer film durability in phosphate-buffered saline (PBS) were assessed (Figure S5, SI). After 24 h in PBS buffer, the release of Ag^+^ ions from the [EC]_n_ and [CA]_n_ biopolymer films doped with 5% of **CP1** and **CP2**, respectively, was below 5 ppm in both films, as attested by the analysis of the solutions by inductively coupled plasma optical emission spectroscopy (ICP-OES). This corresponds to less than 1% of the total silver content incorporated into each doped biopolymer. Considering very low operating concentrations of Ag^+^ ions (<5 ppm), the obtained biopolymers function significantly below the toxicity limit of silver.^25,27,30^

The produced bioCPs exhibit limited solubility in aqueous media, which impacts their distribution within the biopolymer films. **CP1** is practically insoluble in PBS and only slightly soluble in water (<2 mg/mL). **CP2** is slightly soluble in PBS and displays good solubility in water (more than 45 mg/mL). This medium-dependent solubility poses challenges for achieving a uniform dispersion of coordination polymers within the films. Nevertheless, unlike discrete soluble complexes or silver salts, the release of silver ions from bioCP-based films is significantly slower, providing these materials with enhanced stability and a greater potential for long-term applications. Both carboxylic acid ligands in **CP1** and **CP2** were selected for their unexplored potential as building blocks for bioCPs and also because of similar hydrolipophilic properties, with log *P* values of 0.67 for Hmsba and 0.60 for KHsba. As a potassium salt, the latter demonstrated superior solubility, even under conditions favorable to bacterial growth, as confirmed by the bacterial growth inhibition halo tests (Figures S27 and S28, SI).

### 2.3 Structures of CP1 and CP2

The coordination polymer **CP1**, [Ag­(msba)]_n_, features a 2D-layer structure (Figure 2). Its asymmetric unit contains a single Ag­(I) center, which adopts a distorted square planar {AgO_3_Ag} environment filled by symmetry-related carboxylate O donors. The observed Ag–O distances are relatively short, with an average of 2.1971(17) Å. A short argentophilic Ag···Ag interaction [2.9121(4) Å] further stabilizes the layer and reinforces the planarity of the coordination motif (Figure 2b). This observation aligns with previous reports describing assemblies of *d* ^10^ silver­(I) centers, typically stabilized by bridging anionic ligands that facilitate Ag···Ag contacts.^42^ The calculated τ_4_ and τ_4_′ values of 0.23 and 0.20, respectively (β = 168.59°, α = 158.57°),^43,44^ confirm a distortion from the ideal square planar geometry and are consistent with other Ag­(I) carboxylate systems displaying argentophilic interactions.^15,45^ These motifs extend into infinite 2D sheets parallel to the *bc* plane. Interlayer association, with layers stacked along the crystallographic *a* axis, is governed primarily by van der Waals interactions between the methylsulfonyl groups, resulting in a close-packed lamellar architecture with negligible solvent-accessible voids (Figure S7, SI). Topological analysis of **CP1** reveals an uninodal 4-connected net, in which each {Ag_2_O} cluster acts as a node and the Hmsba ligand serves as a linker (Figure S8). The resulting 2D network exhibits a square lattice (sql) topology and is characterized by the regular alternation of {Ag_2_O} clusters and linkers.

**Figure 2:**
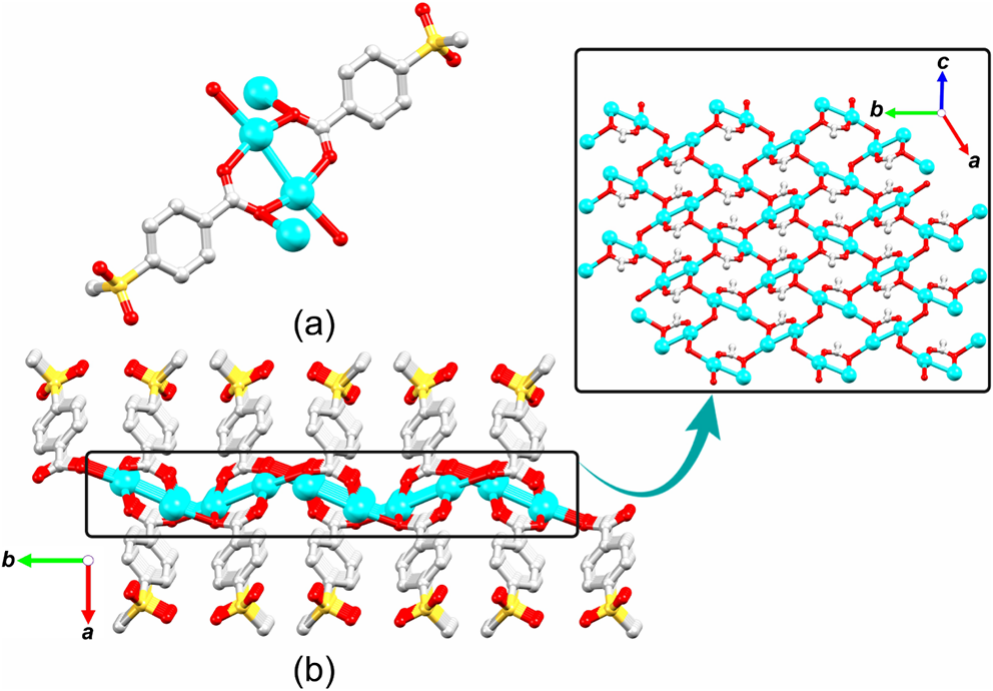
Structure of **CP1**, [Ag­(msba)]_n_. (a) Ligand connectivity. (b) 2D sheets extending parallel to the *bc* plane. Inset: view perpendicular to the *a* axis, with selected ligand atoms omitted for clarity.

The structure of [Ag­(Ksba)­(H_2_O)_2_·2H_2_O]_n_ (**CP2**) features a 3D metal–organic network (Figure 3), wherein each Ag­(I) center adopts a distorted square pyramidal {AgO_3_Ag_2_} environment, with the τ_5_ parameter equal to 0.08 (β = 165.38, α = 160.79°).^44,46^ The silver­(I) center is surrounded by three carboxylate oxygen atoms in a bridging mode, along with two Ag···Ag interactions [2.8274(15) and 3.200(2) Å], which further stabilize the network composed of double ladder-type chain motifs parallel to the *a* axis. Between these silver-based 1D motifs, the 2D layers comprising potassium cations and water molecules are intercalated and multiply bound to sulfonate groups of sba^2–^ (average K–O distance of 2.704(8) Å) into 2D layer motifs with a hexagonal lattice (hxl) topology (Figure S11). The alternating arrangement of 1D silver-based chain motifs and 2D K/H_2_O layers gives rise to an intricate 3D metal–organic framework with a pronounced ionic character.

**Figure 3:**
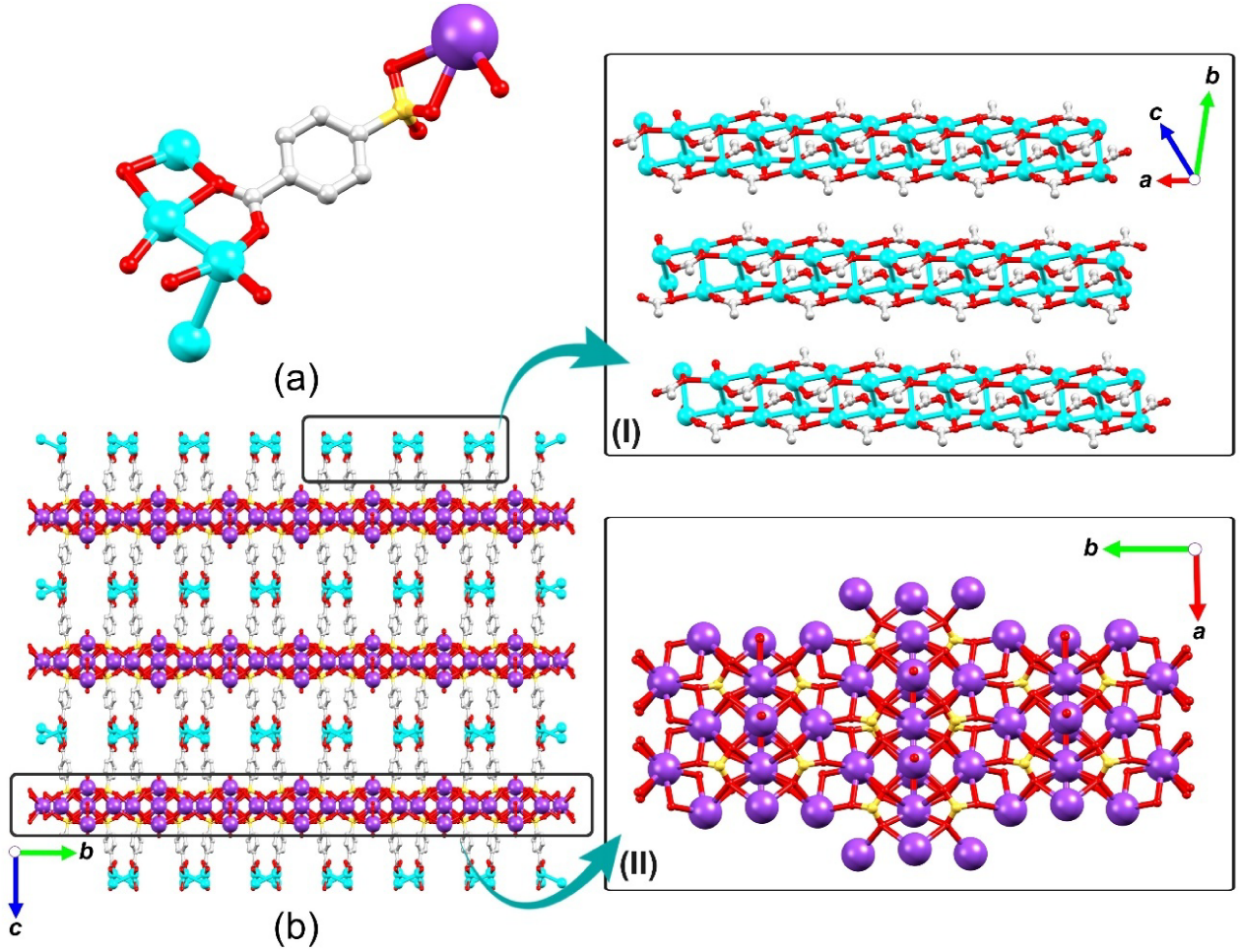
Structure of **CP2**, [Ag­(Ksba)­(H_2_O)_2_·2H_2_O]_n_. (a) Ligand connectivity. (b) View of the 3D pillared framework along the *bc* plane. Inset: (I) 1D silver-based ladder-type motifs; (II) intercalated 2D K/H_2_O layer motifs.

The unit-cell volume of **CP2** is significantly larger than that of **CP1**, and a direct comparison reveals various structural distinctions. Small modifications in the ligand and incorporation of additional metal cations (K^+^) may influence the physicochemical and bioactive properties.^47–49^ **CP1** relies on carboxylate bridges and argentophilic contacts to extend into 2D planar sheets (Figure 3), whereas **CP2** is driven by both sulfonate groups and K/H_2_O moieties to achieve an overall 3D connectivity. When normalized per formula unit (V/Z), **CP1** has a 223.16 Å^3^ volume, while **CP2** expands to 286.19 Å^3^ per asymmetric unit, reflecting a ∼28% increase. This volumetric expansion is attributed to the pillaring effect of K^+^ ions and water molecules (Figure 4), which introduce additional steric bulk and generate discrete solvent-filled voids (378 Å^3^ per unit cell, Table S6, Figure S10). These structural characteristics contribute to an increased solubility of the compound in water,^50^ as observed for **CP2**, while limiting solubility in PBS, which is consistent with the experimental solubility behavior. The distinct architectures of the 2D and 3D silver­(I) CPs give rise to different modes of silver-ion release and surface interaction (Figure S11), with direct implications for their antimicrobial performance.

**Figure 4:**
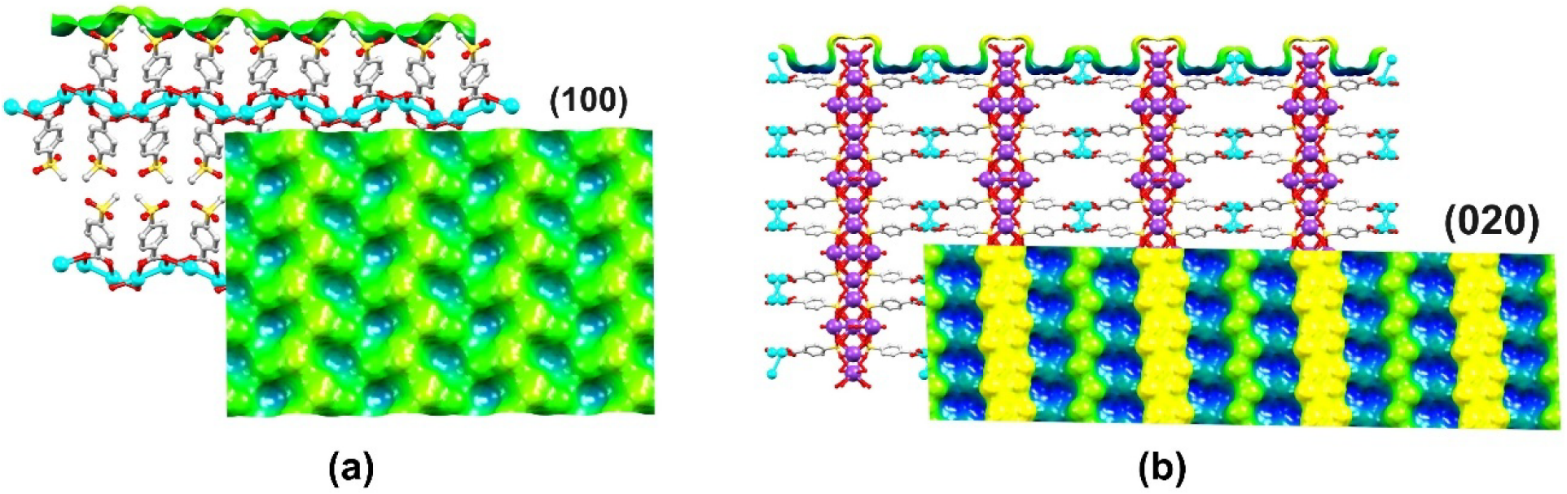
Surface representations with the predominant interactions. (a) Crystallographic (100) plane of **CP1**, exposing terminal −SO_2_CH_3_ groups (hydrophobic interface). (b) Crystallographic (020) plane of **CP2**, showing exposed K^+^ ions and Ag···Ag interactions (hydrophilic interface). For details, see the SI.

### 2.4 Powder X-ray Diffraction

The main diffraction peaks observed in the experimental patterns of **CP1** and **CP2** show correspondence in 2θ positions with those of the simulated diffractograms, indicating that the samples predominantly consist of the targeted crystalline phase (Figures S14–S16). To further characterize the samples, the crystallite sizes were estimated using the Scherrer equation,^51^ assuming a shape factor (K) of 0.9, which corresponds to spherical crystallites. The most intense peaks for **CP1** were at 2θ of 5.88° and 11.81°, which correspond to the (100) and (200) reflections. The calculated average size of the crystallites was approximately 161.79 ± 22.69 nm. For the compound **CP2**, the peaks at 2θ of 9.88°, 12.43°, and 13.08° are attributed to the (201), (022), and (003) reflections, which resulted in an estimated crystallite size of 77.72 ± 19.07 nm. The first two peaks of **CP2** were excluded from the crystallite size calculation due to peak overlap, which could interfere with the accurate size estimation. These findings indicate that both compounds consist of nanoscale crystalline domains. In addition, the PXRD data support the nanocrystalline nature of the synthesized materials.^52^

### 2.5 Morphological Characterization of Biopolymer Films

The bioCP-doped [EC]_n_ and [CA]_n_ films were analyzed by SEM-EDX (Figures 5, 6, and S17–S22) to assess their morphology and the incorporation of **CP1** or **CP2**. The two films, [EC]_n_ and [CA]_n_, exhibit morphologies as illustrated in Figures 5a and 6a. The surface of the [CA]_n_ films shows an increased smoothness and uniformity in comparison to that of [EC]_n_. Figures 5b and 5c depict the surfaces of the [EC]_n_ matrix doped with **CP1** and **CP2**, respectively. The dopants induce markedly distinct morphologies on the surface of [EC]_n_ films, characterized by the presence of CP agglomerates throughout the surface, and this fact is even more notable for the [CA]_n_ matrix (Figure 5b,c). While **CP1** in the [CA]_n_ matrix led to a considerably homogeneous film, **CP2** remained close to the surface of [CA]_n_ forming prolonged crystals with a parallelepiped shape (Figure 6c,g). A rougher surface may have contributed to the creation of these crystals.

**Figure 5:**
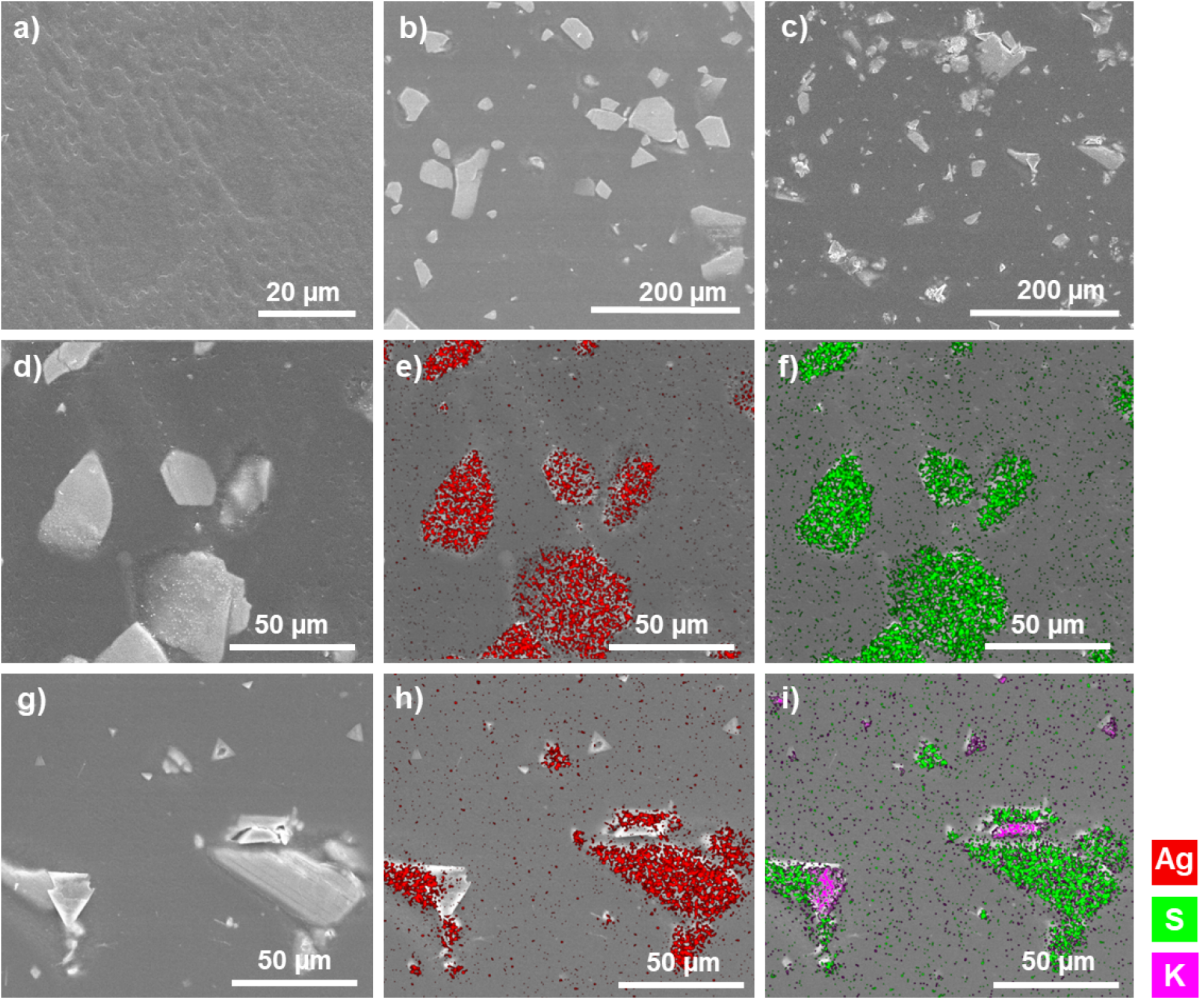
Morphological characterization of [EC]_n_ films by SEM-EDX. SEM images: (a) [EC]_n_ film; (b) **CP1** ^(5%)^@[EC]_n_; (c) **CP2** ^(5%)^@[EC]_n_ [the same region as (b)] with EDX analysis of Ag distribution; (d) **CP2** ^(5%)^@[EC]_n_, where bioCP particles can be seen throughout the material; (e) **CP2** ^(5%)^@[EC]_n_ with EDX analysis of Ag distribution; (f) **CP2** ^(5%)^@[EC]_n_ (the same region as d) with EDX analysis of S distribution; (g) [EC]_n_ film; (h) **CP1** ^(5%)^@[EC]_n_, where bioCP particles can be seen throughout the material; (i) **CP1** ^(5%)^@[EC]_n_ (the same region as g) with EDX analysis of Ag distribution. Magnification: (a) 1000×, (b,c) 150×, and (d,e,f,g,h,i) 500×.

**Figure 6:**
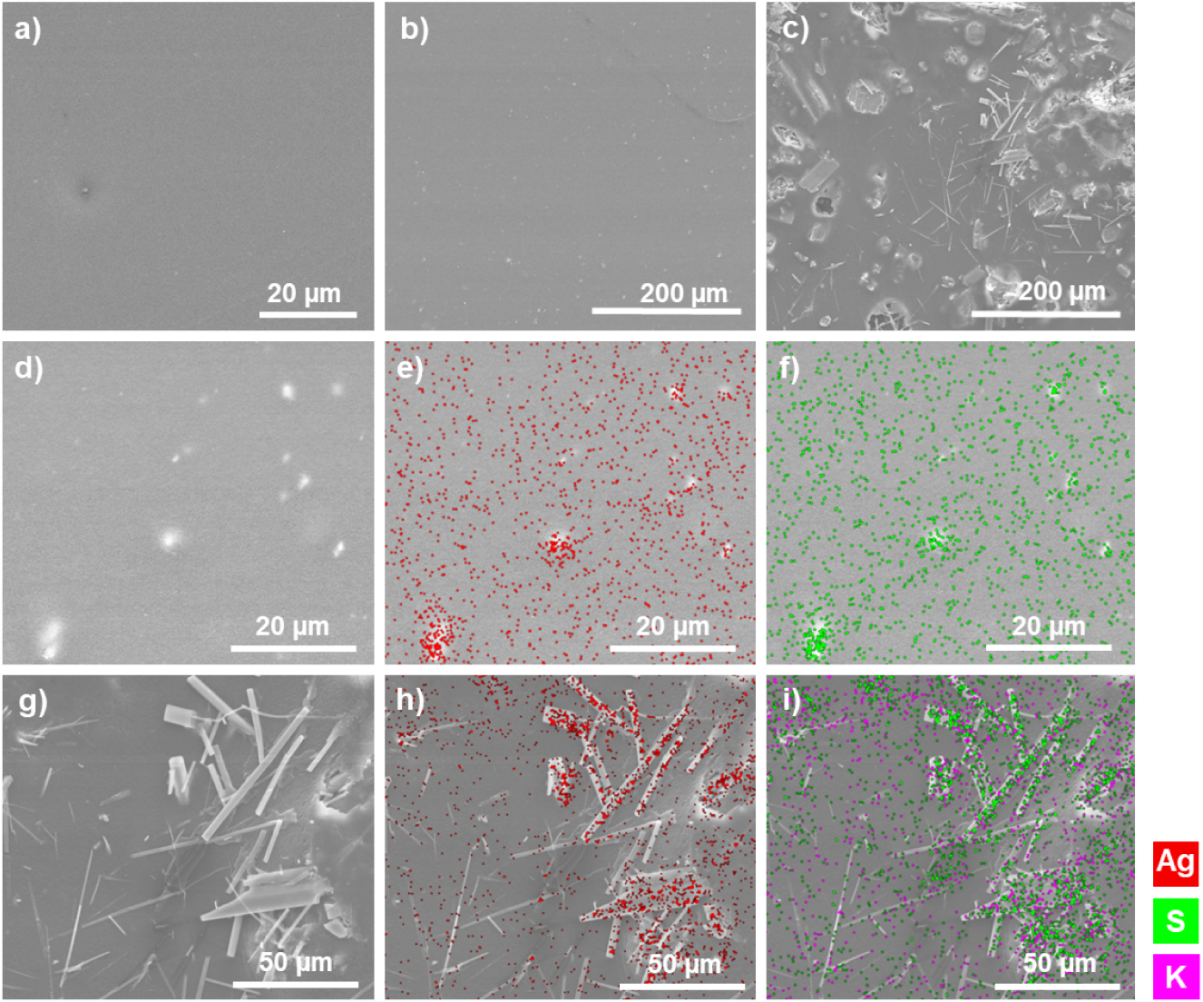
Morphological characterization of [CA]_n_ films by SEM-EDX. SEM images: (a) [CA]_n_ film; (b) **CP1** ^(5%)^@[CA]_n_; (c) **CP2** ^(5%)^@[CA]_n_ [the same region as (b)] with EDX analysis of Ag distribution; (d) **CP2** ^(5%)^@[CA]_n_, where bioCP particles can be seen throughout the material; (e) **CP2** ^(5%)^@[CA]_n_ with EDX analysis of Ag distribution; (f) **CP2** ^(5%)^@[CA]_n_ (the same region as e) with EDX analysis of S distribution; (g) [CA]_n_ film; (h) **CP1** ^(5%)^@[CA]_n_, where bioCP particles can be seen throughout the material; (i) **CP1** ^(5%)^@[CA]_n_ (the same region as g) with EDX analysis of Ag distribution. Magnification: (a,d,e,f) 1000×, (b,c) 150×, (g,h,i) 500×.

It should be noted that ultrasonication and stirring were employed during film preparation to minimize the formation of agglomerates associated with the insoluble nature of the coordination polymers. Although some localized aggregates were still observed in the cross-sectional analysis (Figure S30, SI), the hybrid films exhibited relatively homogeneous dispersion of the CPs throughout the polymer matrices. Importantly, the presence of occasional agglomerates did not affect the reproducibility of the antimicrobial assays, which were performed in triplicate using three independent 10 mm biopolymer discs.

### 2.6 Mechanical Properties of Biopolymer Films

The effects of different plasticizers (glycerol, sorbitol, and polyethylene glycol) on cellulose acetate and ethyl cellulose films were studied. Transparent and flexible films were obtained for all studied combinations, and their mechanical properties were evaluated (Figure 7), namely tensile strength (TS), percentage of elongation at break (EB), and Young’s modulus (YM).

**Figure 7:**
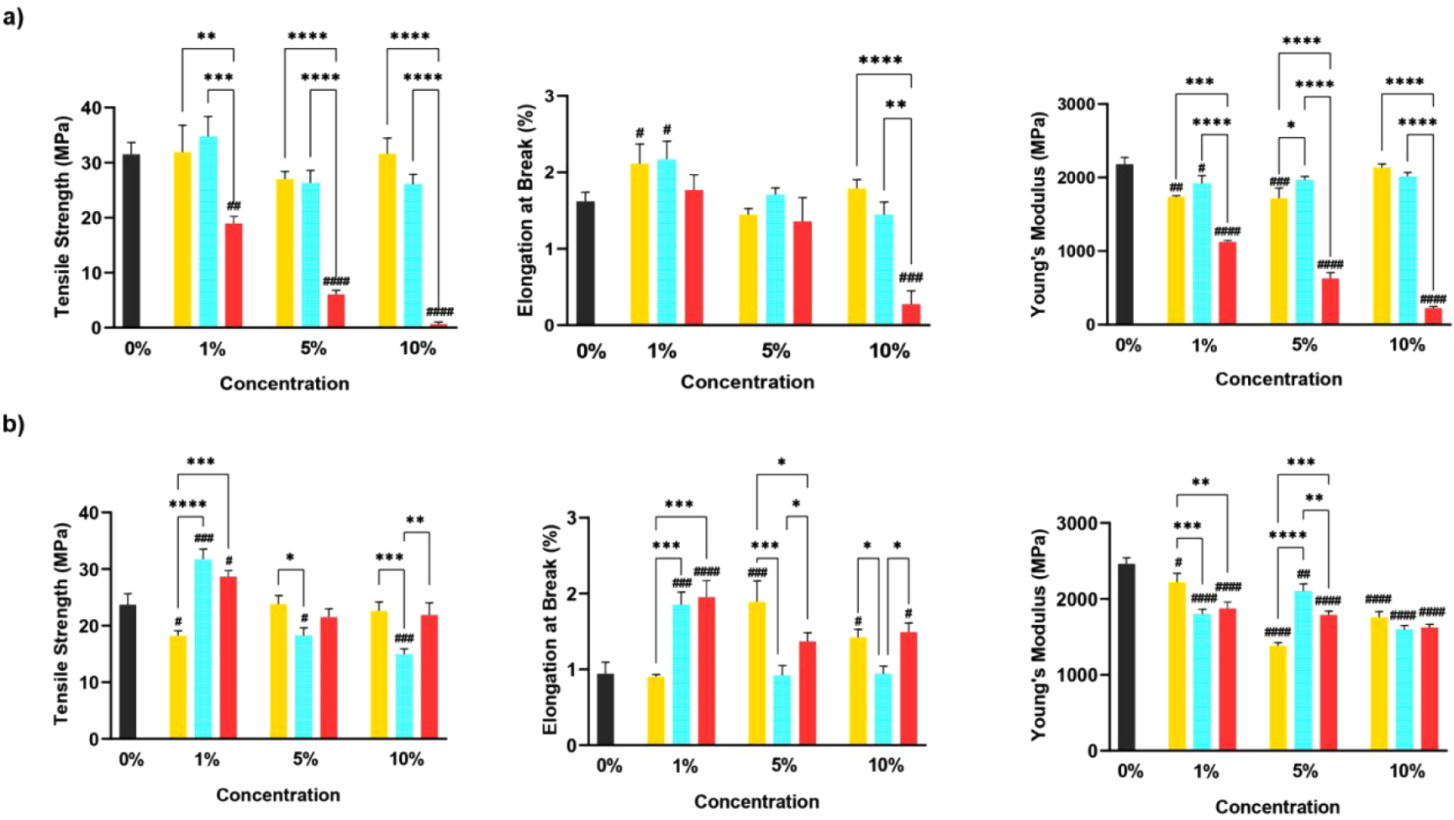
Mechanical properties of: (a) [CA]_n_ and (b) [EC]_n_ biopolymer films obtained with 0%, 1%, 5%, and 10% of the plasticizers: glycerol (yellow), PEG (cyan), and sorbitol (red); without plasticizer (black). Results are depicted as the mean with error bars representing the SEM. Significant statistical differences were calculated comparing all datasets with the control (#) and different plasticizers at the same concentration (*): *P* < 0.05 (*,#), *P* < 0.01 (*,##), *P* < 0.001 (*,###), *P* < 0.0001 (*,####).

The results of TS tests showed that [CA]_n_ films (31.5 MPa) were slightly more robust than [EC]_n_ (23.8 MPa). Both materials can be considered relatively strong when compared to low-density polyethylene (∼10 MPa) or starch (4–20 MPa)^53^ and, consequently, can be suitable for applications where robustness is particularly critical, such as in packaging, coatings, or biomedical uses. The addition of 5% and 10% plasticizer generally resulted in a decrease in TS, while the effect of 1% plasticizer varied depending on its type. However, when it comes to Young’s modulus, the addition of plasticizers consistently lowers this value, leading to reduced film stiffness. The [EC]_n_ films are especially sensitive to the presence of sorbitol.

The EB results for [CA]_n_ and [EC]_n_ films revealed typical values for materials with high crystallinity and low ductility, suggesting that both could benefit from the addition of plasticizers to enhance flexibility. Overall, the introduction of 1% PEG to cellulose acetate proved to be the most promising combination to obtain a more elastic and plastic film with a TS of 34.78 MPa and an EB of 2.17%. A similar behavior was observed on the [EC]_n_ films, where the addition of 1% PEG increased strength from 23.73 to 31.75 MPa and elongation from 0.94 to 1.85%. On the basis of these results, the addition of 1% PEG to [CA]_n_ and [EC]_n_ films was selected for producing the bioCP-doped films.

### 2.7 Water Absorption by Biopolymer Films

The water absorption results (Figure S5) show that the swelling capacity was slightly higher for the [CA]_n_ films (11.76%) compared to that of [EC]_n_ (5.72%), indicating that [CA]_n_ has a better ability to absorb water and swell. This difference is consistent with the structural characteristics of biopolymers. Both materials are derived from cellulose, with the [CA]_n_ films containing acetate groups (−OCOCH_3_) and [EC]_n_ films having apolar ethyl groups (−C_2_H_5_). The presence of the hydrophilic acetate groups in [CA]_n_ enhances water absorption, while the apolar ethyl moiety in [EC]_n_ reduces its affinity for water, rendering the latter less hydrophilic. Overall, the incorporation of 5% **CP1** or **CP2** into the studied matrices did not result in significant changes in the swelling behavior. The doped films exhibited swelling capacities comparable to those of the corresponding control films, suggesting that at this concentration, the presence of coordination polymers does not substantially influence water uptake. Therefore, the swelling properties are mainly governed by intrinsic structural characteristics of the biopolymer matrices.

### 2.8 Thermogravimetric Analysis

Thermal stability of [EC]_n_ and [CA]_n_ films was assessed by thermogravimetric analysis (TGA) under nitrogen atmosphere. TGA data (Figures S6–S9) show that both [EC]_n_ and [CA]_n_ films exhibit similar thermal degradation profiles. Initially, a gradual weight loss of about 10% is observed up to 300 °C, attributed to the release of volatile materials and to thermal expansion. Between 300 and 400 °C, a significant increase in weight loss occurs, with [CA]_n_ and [EC]_n_ films losing approximately 75% and 85% of their weight, respectively. After reaching 400 °C, a slower and more gradual weight loss of about 10% continues between 400 and 800 °C.

Doped **CP1** ^(5%)^@[EC]_n_ and **CP1** ^(5%)^@[CA]_n_ films displayed similar thermal degradation profiles, with their thermograms shifted to slightly higher temperatures compared to the unmodified films. In contrast, **CP2** ^(5%)^@[EC]_n_ and **CP2** ^(5%)^@[CA]_n_ films exhibited profiles shifted to lower temperatures. Specifically, **CP1** showed a profile similar to that of unmodified films, while **CP2** displayed a broader and more gradual mass loss between 250 and 800 °C, ultimately losing 42% of its weight. This suggests that the incorporation of **CP2** influences the thermal stability of the hybrid films in a more pronounced way compared to **CP1**.

### 2.9 Antibacterial Activity

Considering a recognized antibacterial activity of different silver-containing materials,^54–59^ the [CA]_n_ and [EC]_n_ biopolymer films were tested against four different bacterial species at varying concentrations of dopant, namely, 1%, 2.5%, and 5% (m/m) of **CP1** or **CP2**. Materials doped with Hmsba (organic ligand control for **CP1**), KHsba (organic ligand control for **CP2**), or AgNO_3_ (positive control) were also screened. Antibacterial activity was measured in an agar diffusion assay, as it is positively correlated with the size of the growth inhibition halo formed around the biopolymer film samples.^9,15^ Concerning the Ag-doped [CA]_n_ films (Figure 8), all of them showed antibacterial activity against the four tested species, with the films doped with **CP1** or **CP2** showing similar or improved activity compared to the AgNO_3_ control against *P. aeruginosa* and *S. aureus*. Although doped films were less effective against *E. coli* and *S. epidermidis*, the activity was equal to or higher than that of the control, with **CP2** at 2.5% and 5% showing the best improved activity against *E. coli* and *S. epidermidis*, respectively.

**Figure 8:**
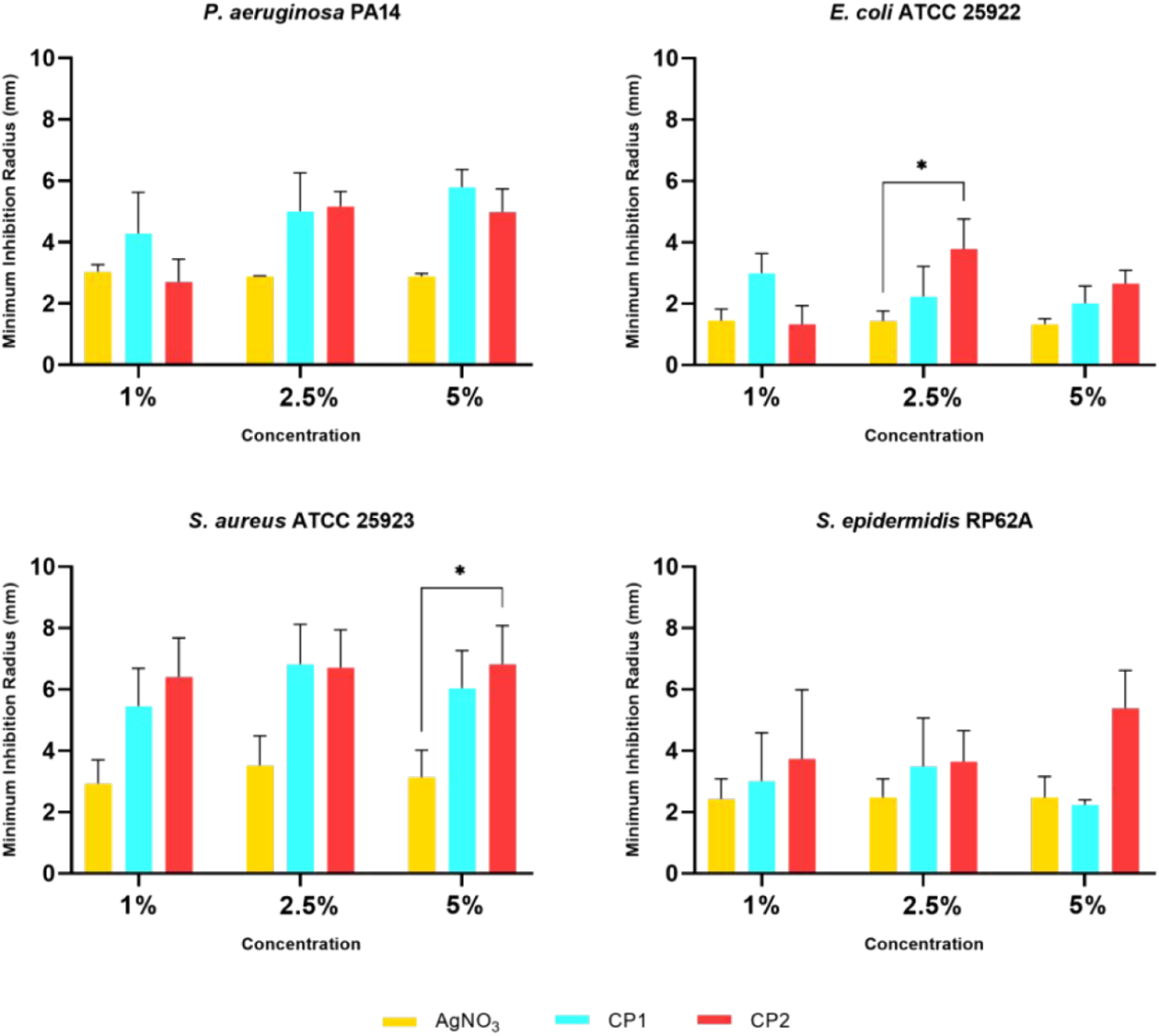
Normalized antibacterial activity of [CA]_n_ films doped with 1, 2.5, and 5% of **CP1**, **CP2**, or AgNO_3_ (positive control) against four bacterial pathogens, namely the Gram-positive *P. aeruginosa* PA14 and *E. coli* ATCC 25922 and the Gram-negative *S. aureus* ATCC 25923 and *S. epidermidis* RP62A. Results are depicted as the minimum radius of the growth inhibition halos with error bars representing the SEM. Significant statistical differences: *P* < 0.05 (*).

Concerning the [EC]_n_ films (Figure 9), the activity of those doped with **CP1** was lower than or similar to that of the AgNO_3_ control. In contrast, films doped with **CP2** were generally more effective than the control against *P. aeruginosa*, *S. aureus*, and *E. coli*, with better activity against the first two strains, consistent with observations for the [CA]_n_ films.

**Figure 9:**
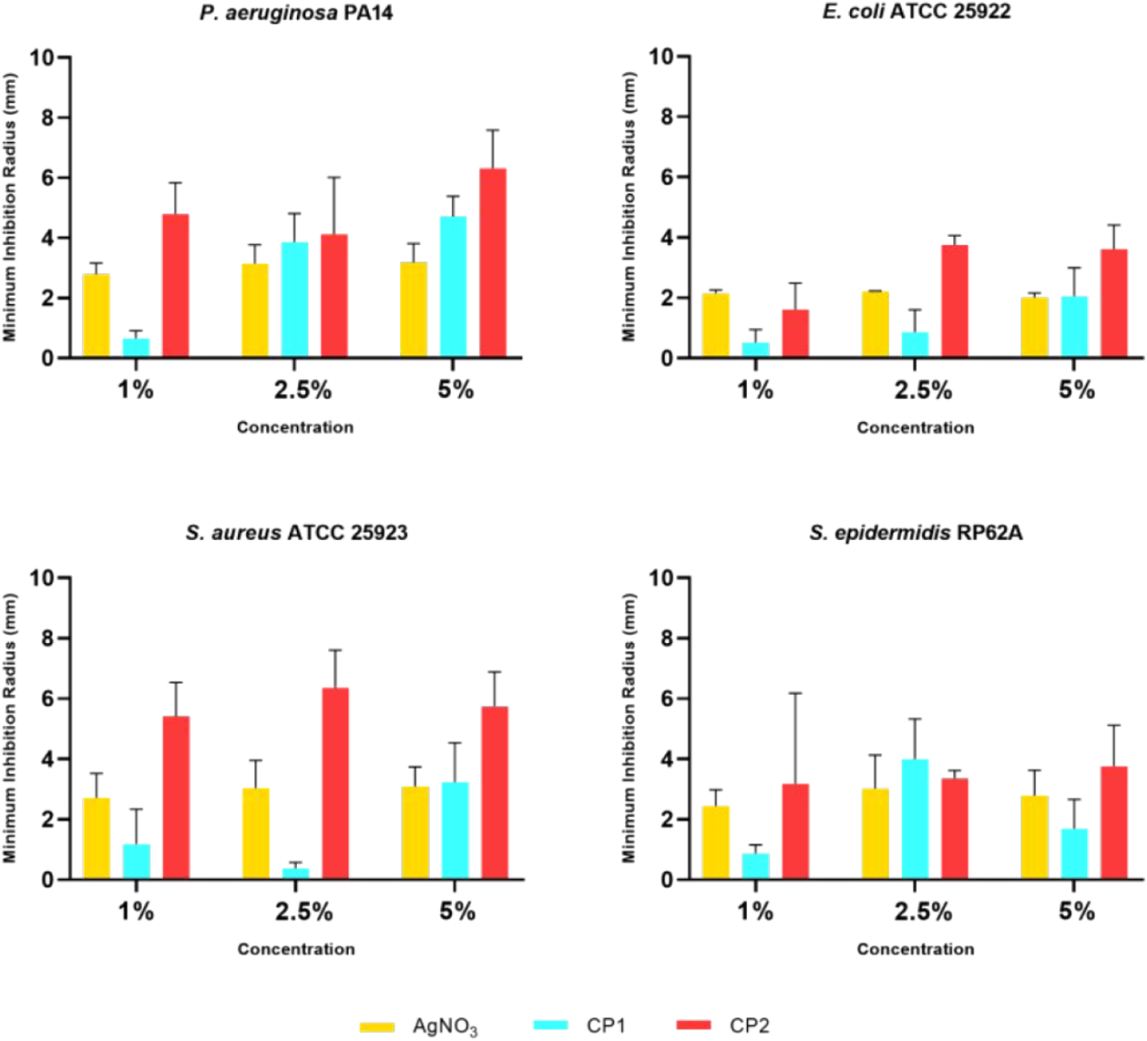
Normalized antibacterial activity of [EC]_n_ films doped with 1, 2.5, and 5% of **CP1**, **CP2**, or AgNO_3_ (positive control) against four bacterial pathogens, namely the Gram-positive *P. aeruginosa* PA14 and *E. coli* ATCC 25922 and the Gram-negative *S. aureus* ATCC 25923 and *S. epidermidis* RP62A. Results are depicted as the minimum radius of the growth inhibition halos with error bars representing the SEM. No significant statistical differences encountered.

Overall, antibacterial activity appears to be independent of dopant concentration and of the type of bacterial cell wall, i.e., the activity was not specifically better against Gram-positive or Gram-negative bacterial species for both [CA]_n_ and [EC]_n_ films. This observation may be explained by saturation of the release mechanism. Even at the lowest dopant concentrations used (1%), the films are likely to release a sufficient amount of silver ions to achieve a maximal antibacterial effect. Increasing the dopant content does not enhance activity, as the rate of release and the local concentration of silver ions at the film surface are the limiting factors. This behavior is further supported by the water absorption and swelling properties of the films, which suggest that the base biopolymer matrix predominantly governs swelling, thereby influencing the diffusion and availability of active species. Consequently, once a threshold concentration of silver ions is reached at the surface, the antibacterial effect is maintained regardless of further increases in dopant loading or differences in bacterial cell wall structure. No activity was observed for doped films with ligands (data not shown). The [CA]_n_ films appear to be a more suitable matrix for **CP1**, as their antibacterial activity was higher than that of the corresponding [EC]_n_ films, particularly against *S. aureus*, where this difference was most pronounced. The [CA]_n_ films have acetate groups (−OCOCH_3_) that make them more hydrophilic, which means they can swell more (11.76%) than the [EC]_n_ films, which are less hydrophilic (3.72%, Figure S5). This increased swelling shows that the [CA]_n_ films can absorb more water, which makes it easier for silver ions or bioCPs to diffuse to the film surface, thereby likely increasing the antibacterial efficacy. The relatively low solubility of **CP1** further highlights the importance of the polymer matrix, as achieving sufficient local concentrations at the surface depends on water-mediated diffusion. Although **CP1** is incorporated in both [CA]_n_ and [EC]_n_ films, the higher water uptake and improved surface availability in [CA]_n_ films likely allow the local silver concentration to more readily exceed the threshold required to inhibit *S. aureus*, making the difference in activity more pronounced for this bacterium. These observations indicate that the base polymer matrix primarily governs water absorption, reinforcing the role of inherent film hydrophilicity in controlling the diffusion and availability of the active species.

### 2.10 Biofilm Inhibition Activity

The samples of Ag-doped [CA]_n_ and [EC]_n_ biopolymer films were also tested against the same bacterial species by immersing them in a bacterial culture for 24 h to assess their capability in preventing bacterial adhesion and biofilm formation on their surface. Biofilm inhibition was determined through quantification of viable adhered cells (CFU counting), expressed as the reduction in adhered cells compared to the negative control (films without dopant, in which biofilms grew in the range of 6–7 log (CFU/cm^2^).^9,15^ Concerning the [CA]_n_ films (Figure 10), both **CP1** and **CP2** were significantly more effective in preventing biofilm formation than the silver control for all tested species, with **CP2** showing the most promising results, reaching reductions up to 3.5 log (99.97% reduction), with *E. coli* being the most susceptible and *S. epidermidis* the least susceptible species. Films doped with the organic ligands did not significantly influence bacterial adhesion, except for *S. aureus*, which was able to show a slight improvement in adhesion up to 1 log.

**Figure 10:**
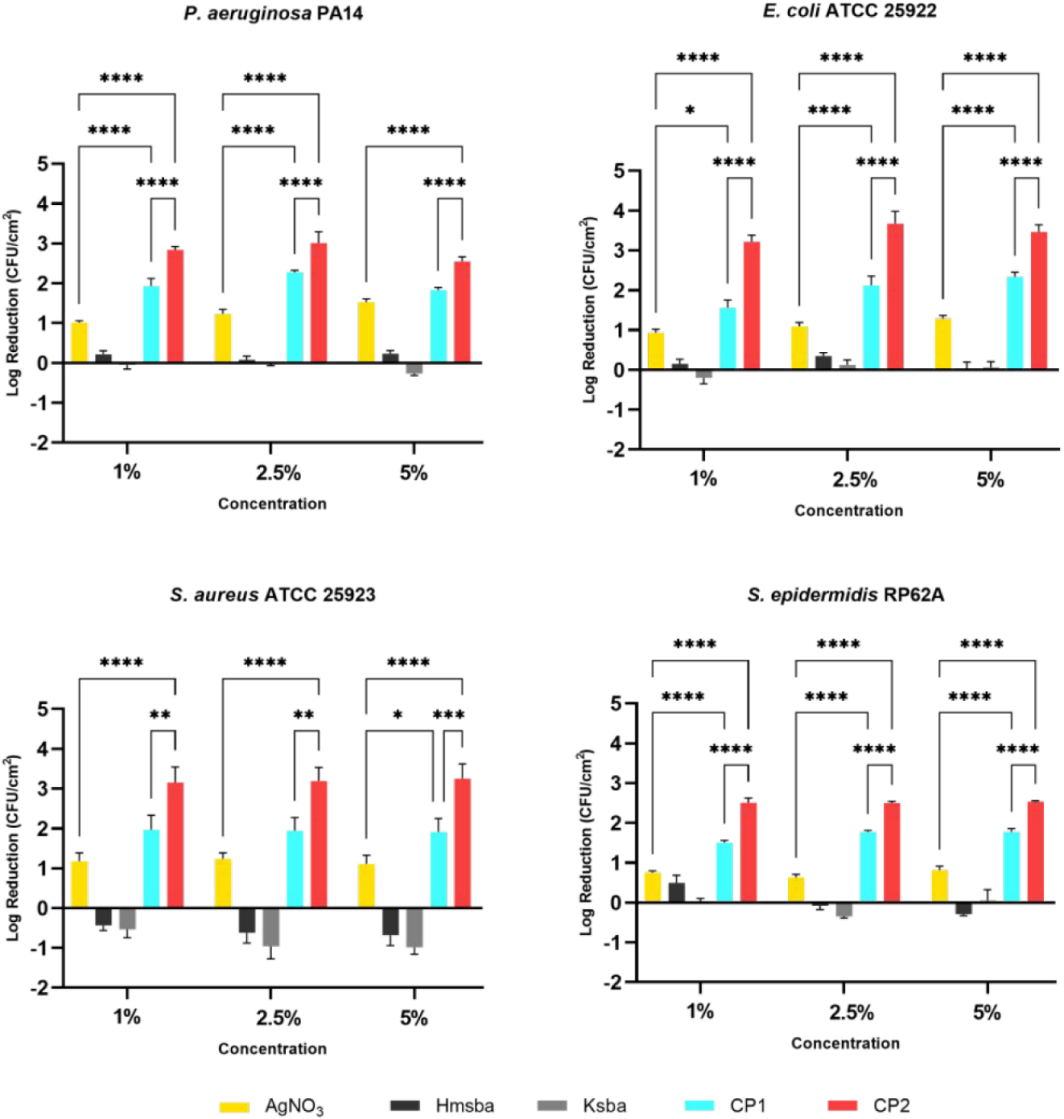
Normalized biofilm inhibition activity of [CA]_n_ films doped with 1, 2.5, and 5% of **CP1**, **CP2**, AgNO_3_ (positive control), Hmsba (organic ligand control for **CP1**), or KHsba (organic ligand control for **CP2**), against four bacterial pathogens, namely the Gram-positive *P. aeruginosa* PA14 and *E. coli* ATCC 25922 and the Gram-negative *S. aureus* ATCC 25923 and *S. epidermidis* RP62A. Results are depicted as log reductions (relative to the nondoped [CA]_n_ matrix control) with error bars representing the SEM. Significant statistical differences: *P* < 0.05 (*), *P* < 0.01 (**), *P* < 0.001 (***), *P* < 0.0001 (****).

Similar observations were made for the [EC]_n_ films (Figure 11), with the additional evidence that the films doped with ligands had an improved positive effect on the adhesion of *S. epidermidis*. As observed in the antibacterial assays (Figures 8 and 9), there is no clear dependency of the effect on concentration as well as no correlation with the bacterial Gram classification. However, in the biofilm assays, it appears that both film matrices showed similar results, contrasting with the observation made in the agar diffusion assays. This behavior may be related to the distinct experimental configurations used in the two assays. In the biofilm setup, the diffusion of active compounds into the medium is facilitated because the sample is fully immersed in the liquid environment rather than being exposed to the medium only on one side, as in the agar-based tests.

**Figure 11:**
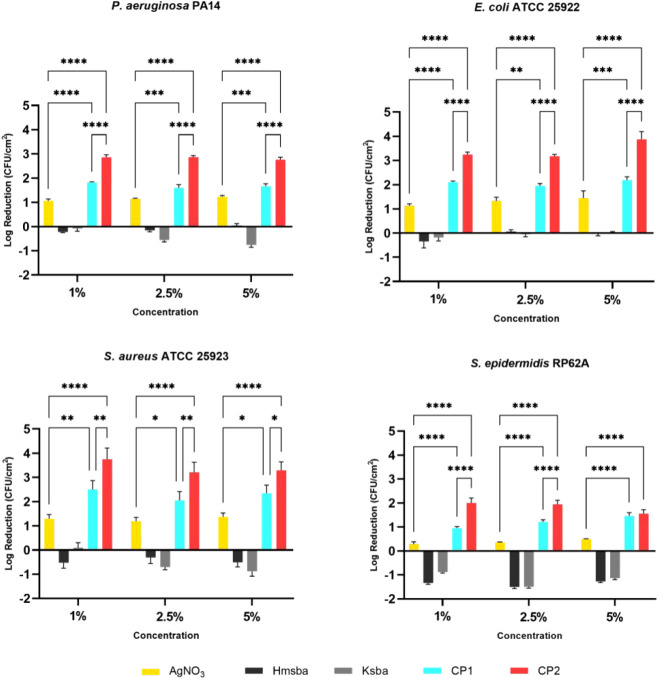
Normalized biofilm inhibition activity of [EC]_n_ films doped with 1, 2.5, and 5% of **CP1**, **CP2**, AgNO_3_ (positive control), Hmsba (organic ligand control for **CP1**), or KHsba (organic ligand control for **CP2**), against four bacterial pathogens, namely the Gram-positive *P. aeruginosa* PA14 and *E. coli* ATCC 25922 and the Gram-negative *S. aureus* ATCC 25923 and *S. epidermidis* RP62A. Results are depicted as log reductions (relative to the nondoped matrix control) with error bars representing the SEM. Significant statistical differences: *P* < 0.05 (*), *P* < 0.01 (**), *P* < 0.001 (***), *P* < 0.0001 (****).

Overall, antibacterial and biofilm inhibition results are consistent with previous reports from our group,^9,15,59^ where silver-containing coordination polymer-doped biopolymer films exhibited strong activity against both Gram-positive and Gram-negative bacteria. In particular, silver-doped polysaccharide films based on sulfonyldibenzoate coordination polymers were previously shown to produce biofilm reductions, reaching complete inhibition in some cases (up to ∼7.5 log reduction),^15^ highlighting the strong potential of silver-containing coordination polymers as antimicrobial dopants for bioactive surfaces. The present results further support the versatility of this strategy across different polymer matrices and related systems.^60–62^

### 2.11 Structure–Activity Relationship and Mechanistic Implications

In **CP1**, the methyl-rich interface is expected to preferentially associate with the thick and porous peptidoglycan layer of Gram-positive organisms (e.g., *Staphylococcus aureus* and *Staphylococcus epidermidis*) via hydrophobic interactions. This interaction might facilitate the anchoring of the compound to the bacterial cell wall, allowing surface-mediated release of Ag^+^ ions over time, which is consistent with bacteriostatic or bactericidal mechanisms reported in the literature.^54,55^ In **CP2**, the cation-terminated surfaces are expected to interact strongly with the anionic lipopolysaccharide (LPS) outer membrane of Gram-negative bacteria (e.g., *Pseudomonas aeruginosa*, *Escherichia coli*) through electrostatic attractions.^56–58^ Additionally, the presence of K^+^ centers in **CP2** increases the hydrophilicity and changes the ionic strength at the interface, which may affect microbial adhesion and uptake pathways. The high local concentration of readily available Ag^+^ ions, along with K^+^, is expected to facilitate a rapid translocation of metal ions across the outer membrane, thus causing a burst of antibacterial activity. Figures 10 and 11 (as well as Figures S27 and S28, SI) illustrate how the contrasting surfaces of **CP1** and **CP2** account for their distinct antimicrobial profiles. In summary, the obtained results show that CP-doped biopolymers are particularly promising materials for inhibiting bacterial adhesion and biofilm formation, especially for biopolymers doped with **CP2**, with reductions up to 3.5 log CFU/cm^2^ (99.97% reduction).

## 3 Experimental Section

### 3.1 Synthesis and Characterization of 1 and 2

#### 3.1.1 Synthesis of CP1

Acetonitrile (10 mL) was added to 4-(methylsulfonyl)­benzoic acid (Hmsba, 0.100 g, 0.5 mmol), followed by the addition of 1.5 mL of aq. NH_4_OH (4 M solution, 6 mmol). Then, AgNO_3_ (0.085 g, 0.5 mmol) was dissolved in water (1 mL), and the obtained solution was introduced to the reaction mixture. This was stirred for 2 h at room temperature and then filtered through a filter paper. The filtrate was left to slowly evaporate in a vial at room temperature. Colorless X-ray quality crystals were collected and air-dried to obtain compound **CP1** in 62% yield, based on silver­(I) nitrate. Anal. Calcd (%) for C_8_H_7_AgO_4_S (**CP1**): C 31.3, H 2.3, S 10.4; found: C 31.3, H 1.7, S 10.6. FTIR-ATR (cm^–1^): 3094 w, 3023 w, 3009 w, 2929 w, 1577 m, 1151 m, 1507 m, 1485 m, 1388 s, 1375 s, 1313 s, 1298 s, 1289 s, 1170 m, 1152 s, 1129 s, 1108 m, 1087 s, 1014 m, 966 m, 959 s, 874 m, 866 w, 854 w, 840 s, 791 s, 752 vs, 719 s, 689 s. ^1^H NMR (DMSO-*d*_6_, 400 MHz): δ, ppm: 8.13 (d, *J* = 8.4 Hz, 2H), 7.92 (d, *J* = 8.4 Hz, 2H), 3.22 (s, 3H).

#### 3.1.2 Synthesis of CP2

Compound **CP2** was synthesized by following the procedure described for **CP1**. A mixture of acetonitrile/water (10/10 mL) was added to 4-sulfobenzoic acid monopotassium salt (KHsba, 0.240 g, 1 mmol), followed by the addition of 5 mL of aq. NH_4_OH (4 M solution, 20 mmol). Then, AgNO_3_ (0.170 g, 1 mmol) was introduced. The reaction mixture was stirred for 30 min at room temperature and then filtered off. The filtrate was left to slowly evaporate in air. Colorless X-ray quality crystals were collected and air-dried in a 31% yield, based on silver­(I) nitrate. Anal. Calcd (%) for C_7_H_12_AgKO_9_S (**CP2**+2H_2_O): C 20.1, H 2.9, S 7.6; found: C 19.6, H 2.1, S 7.1. FTIR-ATR (cm^–1^): 3192 br, 1601 s, 1555 s, 1385 s, 1217 s, 1201 s, 1146 m, 1119 s, 1083 w, 1040 s, 1009 s, 835 m, 826 m, 792 s, 740 vs, 700 s. ^1^H NMR (DMSO-*d*_6_, 400 MHz): δ, ppm: 7.87 (d, *J* = 8.0 Hz, 2H), 7.61 (d, *J* = 8.0 Hz, 2H).

### 3.2 Synthesis of BioCP-Doped [EC]_n_ and [CA]_n_ Films

Before incorporation into the [EC]_n_ and [CA]_n_-based biopolymer films, **CP1** and **CP2** were mechanically ground to obtain fine powdered solids. A mixture of EC (2 g), ethanol (20 mL), and PEG 1000 (0.02 g) (1:10:0.01 mass ratio) was prepared by mixing EC powder in ethanol at 80 °C until complete dissolution, followed by the addition of plasticizer. Then, the compounds **CP1** or **CP2** were introduced into the EC-based mixtures, ultrasonically treated for 5 min, and afterward stirred at 60 °C for 15 min until reaching complete homogenization. The mixtures were poured into Petri dishes (0.1 m in diameter) and dried at 30 °C in an oven for polymerization for 24 h. The obtained films were abbreviated as **CP1** ^(1%)^@[EC]_n_, **CP1** ^(2.5%)^@[EC]_n_, **CP1** ^(5%)^@[EC]_n_, **CP2** ^(1%)^@[EC]_n_, **CP2** ^(2.5%)^@[EC]_n_, and **CP2** ^(5%)^@[EC]_n_. FTIR-ATR (cm^–1^): 2973 m, 2864 br, 1374 w, 1355 m, 1313 w, 1277 w, 1049 vs, 919 m, 881 m.

For the preparation of [CA]_n_-based biopolymer films, the mixture of cellulose acetate (2 g), acetone (20 mL), and PEG (0.02 g) (1:10:0.01 mass ratio) was prepared by mixing CA powder in acetone at room temperature until complete dissolution before adding the plasticizer. Then, the compounds **CP1** or **CP2** were introduced, and the obtained mixtures were ultrasonically treated for 5 min and afterward stirred for 15 min until reaching complete homogenization. The mixtures were poured into Petri dishes (0.1 m in diameter) and kept at room temperature in a desiccator for 24 h. The obtained films were abbreviated as **CP1** ^(1%)^@[CA]_n_, **CP1** ^(2.5%)^@[CA]_n_, **CP1** ^(5%)^@[CA]_n_, **CP2** ^(1%)^@[CA]_n_, **CP2** ^(2.5%)^@[CA]_n_, and **CP2** ^(5%)^@[CA]_n_. FTIR-ATR (cm^–1^): 1734 vs, 1435 w, 1368 s, 1217 vs, 1162 w, 1031 vs, 899 m.

In parallel, the films for control tests were prepared, namely, [EC]_n_, AgNO_3_^(1%)^@[EC]_n_, AgNO_3_^(2.5%)^@[EC]_n_, AgNO_3_^(5%)^@[EC]_n_, Hmsba^(1%)^@[EC]_n_, Hmsba^(2.5%)^@[EC]_n_, Hmsba^(5%)^@[EC]_n_, Hsba^(1%)^@[EC]_n_, Hsba^(2.5%)^@[EC]_n_, Hsba^(5%)^@[EC]_n_, [CA]_n_, AgNO_3_^(1%)^@[CA]_n_, AgNO_3_^(2.5%)^@[CA]_n_, AgNO_3_^(5%)^@[CA]_n_, Hmsba^(1%)^@[CA]_n_, Hmsba^(2.5%)^@[CA]_n_, Hmsba^(5%)^@[CA]_n_, Hsba^(1%)^@[CA]_n_, Hsba^(2.5%)^@[CA]_n_, and Hsba^(5%)^@[CA]_n_.

Silver-containing materials may exhibit photosensitivity and gradual darkening due to the formation of metallic silver species upon light exposure. However, UV–vis analysis showed no significant changes in film transparency after 1 week of sunlight exposure (Figure S29, SI). As some gradual darkening was observed after prolonged storage, the materials were usually stored in the dark to minimize the light-induced changes.

### 3.3 Antibacterial and Biofilm Inhibition Activity

The antibacterial properties of the biopolymer films doped with **CP1** and **CP2** were assessed in a soft agar overlay assay.^9,15,59^ Briefly, two Gram-negative (*Escherichia coli* ATCC 25922 and *Pseudomonas aeruginosa* PA14) and two Gram-positive (*Staphylococcus aureus* ATCC 25923 and *Staphylococcus epidermidis* RP62A) bacterial strains, known for their prominent role in biomaterial-related infections, were grown in cation-adjusted Mueller–Hinton broth (MHB) overnight and inoculated into soft Mueller–Hinton agar (MHA) (0.5% agar) at a concentration of 1 × 10^6^ CFU mL^–1^. Then, 3 mL of the inoculated soft agar was placed in a 9 cm diameter Petri dish containing 10 mL of solidified hard MHA (1.7% agar). The films were placed on top of the soft MHA and incubated aerobically for about 24 h at 37 °C. Antibacterial activity was assessed by measuring the bacterial growth inhibition halo, more specifically, the minimum growth inhibition radius. Measurements were performed on photographs taken with a Bio-Rad ChemiDoc Imager and using the image editing software GIMP.

Biofilm inhibition of biopolymer films was assessed by viable adhered bacteria quantification (CFU counting) after 24 h incubation, following previously established methodologies.^9,15,59^ Dye-based assays were not employed due to potential interactions between the materials and dyes, which could affect absorbance measurements. Therefore, CFU enumeration was selected as a direct and quantitative method to evaluate bacterial adhesion and biofilm formation. Briefly, bacteria grown overnight in Tryptic Soy Broth (TSB) were transferred to fresh medium at a final concentration of 1 × 10^6^ CFU mL^–1^. The films were placed on the bottom of a 48-well microtiter plate, to which 300 μL of bacterial suspension was added and incubated for 24 h. Afterward, the films were placed in clean wells and washed twice with 0.9% NaCl to remove any nonadhered cells. Adhered bacteria were quantified by first detaching them into 300 μL of 0.9% NaCl in an ultrasonic bath (220 V, 50/60 Hz) for 15 min, followed by 30 s of vortexing. Finally, the detached bacteria were serially diluted in 0.9% NaCl, plated in plates of TSB supplemented with 1.7% agar, incubated overnight, quantified by CFU counting, and presented as the logarithmic reduction of the number of bacteria per cm^2^ of film compared with the controls.

All data were normalized for the molar content of silver­(I) and analyzed in GraphPad Prism 9.3.0 using one-way ANOVA followed by uncorrected Fisher’s Least Significant Difference (LSD) multiple comparisons test with a 95% confidence interval. Data were plotted as the mean along with the standard error of the mean (SEM).

## 4 Conclusions

In this study, 4-(methylsulfonyl)­benzoic acid (Hmsba) and 4-sulfobenzoic acid monopotassium salt (KHsba) were used as promising and still little-explored sulfur-containing carboxylate linkers for the self-assembly of new silver­(I) coordination polymers. The obtained [Ag­(msba)]_n_ (**CP1**) and [Ag­(Ksba)­(H_2_O)_2_·2H_2_O]_n_ (**CP2**) compounds feature distinct 2D and 3D networks, respectively.

These compounds were employed as antibacterial dopants to produce hybrid Ag-doped biopolymer films made of ethylcellulose or cellulose acetate. The films doped with **CP1** or **CP2** revealed a significantly superior antibacterial activity than the positive control samples with AgNO_3_ as a dopant, especially in preventing bacterial adhesion and biofilm formation, with reductions in adhered cells up to 3.5 log CFU cm^2^, which corresponds to a 99.97% decrease in biofilm growth. **CP2** performed better than **CP1**, probably due to the presence of K^+^ ions, which made the compound more hydrophilic and changed the ionic strength at the interface. This could affect the pathways of microbial adherence. For the biofilm inhibition tests, both types of materials based on [CA]_n_ and [EC]_n_ showed similar results. Given their wide-spectrum activity and effectiveness, **CP1**@[CA]_n_, **CP1**@[EC]_n_, and especially **CP2**@[CA]_n_ and **CP2**@[EC]_n_ hold promise as candidates for coating strategies to prevent infections related to surface contamination.

One of the concepts explored in this work was the use of two model biopolymers with different permeabilities and stabilities to generate applied materials. [EC]_n_ has a hydrophobic and less-polarized biopolymer surface, whereas [CA]_n_ features a more polarized surface. Both biopolymers were doped with low amounts of silver­(I) bioCPs to permit the release of antibacterial components in a controlled manner. These materials can thus be considered promising candidates for antimicrobial applications in healthcare, agriculture, and packaging industries. The present study also advances the unexplored biofilm inhibition applications of bioCPs and their derived functional materials.

## Supplementary Material



## Notes

Materials and methods; FTIR-ATR spectra (Figures S1–S4); swelling ability of [EC]_n_ and [CA]_n_ biopolymers (Figure S5); TGA data (Figures S6–S9); crystal data and structure refinement for **CP1** and **CP2** (Table S1); additional structural details (Tables S3–S6) and images (Figures S10–S13) for **CP1** and **CP2**; PXRD patterns (Figures S14–S16); SEM-EDS data (Figures S17–S22, S30); mechanical analysis details and properties (Tables S7–S8); additional antimicrobial data (Figures S23–S28); UV–vis spectra (Figure S29); and crystallographic data in CIF format (CCDC Numbers 2502323–2502324) (PDF)

## References

[1] Xie Y., Liu H., Teng Z., Ma J., Liu G. (2025). Nanomaterial-Enabled
Anti-Biofilm Strategies: New Opportunities for Treatment of Bacterial
Infections. Nanoscale.

[2] Upadhyay A., Jaiswal N., Kumar A. (2025). Biofilm Battle:
New Transformative
Tactics to Tackle Bacterial Biofilm Infections. Microb. Pathog..

[3] Choi V., Rohn J. L., Stoodley P., Carugo D., Stride E. (2023). Drug Delivery
Strategies for Antibiofilm Therapy. Nat. Rev.
Microbiol..

[4] Yin W., Xu S., Wang Y., Zhang Y., Chou S.-H., Galperin M. Y., He J. (2021). Ways to Control Harmful Biofilms: Prevention, Inhibition, and Eradication. Crit. Rev. Microbiol..

[5] Wang C., Wei X., Zhong L., Chan C.-L., Li H., Sun H. (2025). Metal-Based
Approaches for the Fight against Antimicrobial Resistance: Mechanism,
Opportunities, and Challenges. J. Am. Chem.
Soc..

[6] Pinto R. M., Soares F. A., Reis S., Nunes C., Van Dijck P. (2020). Innovative
Strategies Toward the Disassembly of the EPS Matrix in Bacterial Biofilms. Front. Microbiol..

[7] Chen B., Dai Y., Yang S., Zhou X., Guan Y., Ding Z., Wang Z., Xu M., Yuwen L., Wang L. (2026). Recent Progress
of Metal-Based Nanozymes for Biomedical Applications. ACS Appl. Mater. Interfaces.

[8] Zigale T. T., Huang R., Wang L., Dong Q., Deng Y., Zeng K., Zhang Z. (2026). Co–N4-Coordinated Single-Atom
Nanozymes with Dual Bactericidal-Regenerative Functions: Overcoming
Tetracycline Resistance through Self-Enhanced Oxidase-Mimicking Catalysis. ACS Appl. Mater. Interfaces.

[9] Fernandes T. A., Costa I. F. M., Jorge P., Sousa A. C., André V., Cerca N., Kirillov A. M. (2021). Silver
(I) Coordination Polymers
Immobilized into Biopolymer Films for Antimicrobial Applications. ACS Appl. Mater. Interfaces.

[10] Ma K., Cheung Y. H., Xie H., Wang X., Evangelopoulos M., Kirlikovali K. O., Su S., Wang X., Mirkin C. A., Xin J. H. (2023). Zirconium-Based
Metal–Organic Frameworks as
Reusable Antibacterial Peroxide Carriers for Protective Textiles. Chem. Mater..

[11] Han D., Liub X., Wu S. (2022). Metal-Organic Framework-Based Antibacterial
Agents and Their Underlying Mechanisms. Chem.
Soc. Rev..

[12] Mondal S., Sahoo R., Behera J., Das M. C. (2024). Advances on Silver-Based
MOFs and/or CPs and Their Composites: Synthesis Strategies and Applications. Coord. Chem. Rev..

[13] Li R., Chen T., Pan X. (2021). Metal-Organic-Framework-Based Materials
for Antimicrobial Applications. ACS Nano.

[14] Cao M., Liu Z., Wang Z., Ding X., Zhang K., Zhao N., Xu F.-J. (2025). Engineering
Metal–Organic Framework System for Light-Free
Antibacterial Photodynamic Therapy. ACS Appl.
Mater. Interfaces.

[15] Fernandes T. A., Macedo F., Cabral R. G., Guiu T., Franco C. H. J., Jorge P., Sousa A. C., André V., Cerca N., Kirillov A. M. (2024). Sulfonyldibenzoate
Coordination Polymers
as Bioactive Dopants for Polysaccharide Films with Antibacterial and
Antibiofilm Properties. RSC Appl. Interfaces.

[16] Pettinari C., Pettinari R., Di Nicola C., Tombesi A., Scuri S., Marchetti F. (2021). Antimicrobial
MOFs. Coord. Chem.
Rev..

[17] Jaros S. W., Guedes da Silva M. F. C., Florek M., Smoleński P., Pombeiro A. J. L., Kirillov A. M. (2016). Silver
(I) 1,3,5-Triaza-7-Phosphaadamantane
Coordination Polymers Driven by Substituted Glutarate and Malonate
Building Blocks: Self-Assembly Synthesis, Structural Features, and
Antimicrobial Properties. Inorg. Chem..

[18] Kim D., Park K. W., Park J. T., Choi I. (2023). Photoactive MOF-Derived
Bimetallic Silver and Cobalt Nanocomposite with Enhanced Antibacterial
Activity. ACS Appl. Mater. Interfaces.

[19] Wang Y., Dong Y., Quan Y., Wackerow S., Abdolvan A., Zolotovskaya S. A., Zhao Q. (2025). Hybrid Antibacterial Surfaces: Combining
Laser-Induced Periodic Surface Structures with Polydopamine-Chitosan-Silver
Nanoparticle Nanocomposite Coating. Adv. Mater.
Interfaces.

[20] Gordienko M. G., Palchikova V. V., Kalenov S. V., Belov A. A., Lyasnikova V. N., Poberezhniy D. Y., Chibisova A. V., Sorokin V. V., Skladnev D. A. (2019). Antimicrobial
Activity of Silver Salt and Silver Nanoparticles in Different Forms
against Microorganisms of Different Taxonomic Groups. J. Hazard. Mater..

[21] Li S., Li Q., Zhang H., Li F., Hu J., Qian J., Wang Y., Zhang J., Wu Z. (2024). Dental Caries Management
with Antibacterial Silver-Doped Prussian Blue Hydrogel by the Combined
Effects of Photothermal Response and Ion Discharge. ACS Appl. Mater. Interfaces.

[22] Gao D., Wang F., Zheng C., Lv B., Ma J. (2025). A Durable
Ag@MOF-545/QCM-Cotton Fabric with “Intelligent Bacteria-Capturing
and Dual Antibacterial” Properties. ACS
Appl. Mater. Interfaces.

[23] Marchetti F., Palmucci J., Pettinari C., Pettinari R., Scuri S., Grappasonni I., Cocchioni M., Amati M., Lelj F., Crispini A. (2016). Linkage Isomerism
in
Silver Acylpyrazolonato Complexes and Correlation with Their Antibacterial
Activity. Inorg. Chem..

[24] Seyedpour S. F., Arabi Shamsabadi A., Khoshhal Salestan S., Dadashi Firouzjaei M., Sharifian Gh M., Rahimpour A., Akbari Afkhami F., Shirzad Kebria M. R., Elliott M. A., Tiraferri A., Sangermano M., Esfahani M. R., Soroush M. (2020). Tailoring the Biocidal
Activity of Novel Silver-Based Metal Azolate Frameworks. ACS Sustainable Chem. Eng..

[25] Eckhardt S., Brunetto P. S., Gagnon J., Priebe M., Giese B., Fromm K. M. (2013). Nanobio Silver:
Its Interactions with Peptides and
Bacteria, and Its Uses in Medicine. Chem. Rev..

[26] Berchel M., Gall T. L., Denis C., Hir S. L., Quentel F., Elléouet C., Montier T., Rueff J.-M., Salaün J.-Y., Haelters J.-P., Hix G. B., Lehn P., Jaffrès P.-A. (2011). A Silver-Based
Metal–Organic Framework Material as a ‘Reservoir’
of Bactericidal Metal Ions. New J. Chem..

[27] Mimura E. C. M., Favoreto J. P. M., Favero M. E., Bonifacio K. L., Peixe T. S., Morita A. A., Barbosa D. S., Yabe M. J. S., Carrilho A. J. F. (2020). Silver Serum Levels in Burned Patients
Treated with Silver Sulfadiazine and Its Toxicity on Inflammatory
Cells. Burns.

[28] Sandri G., Bonferoni M. C., Ferrari F., Rossi S., Aguzzi C., Mori M., Grisoli P., Cerezo P., Tenci M., Viseras C., Caramella C. (2014). Montmorillonite–Chitosan–Silver
Sulfadiazine Nanocomposites for Topical Treatment of Chronic Skin
Lesions: In Vitro Biocompatibility, Antibacterial Efficacy and Gap
Closure Cell Motility Properties. Carbohydr.
Polym..

[29] Fajardo A. R., Lopes L. C., Caleare A. O., Britta E. A., Nakamura C. V., Rubira A. F., Muniz E. C. (2013). Silver Sulfadiazine
Loaded Chitosan/Chondroitin
Sulfate Films for a Potential Wound Dressing Application. Mater. Sci. Eng., C.

[30] Medici S., Peana M., Crisponi G., Nurchi V. M., Lachowicz J. I., Remelli M., Zoroddu M. A. (2016). Silver
Coordination Compounds: A
New Horizon in Medicine. Coord. Chem. Rev..

[31] Malekjani N., Jafari S. M. (2021). Modeling the Release of Food Bioactive Ingredients
from Carriers/Nanocarriers by the Empirical, Semiempirical, and Mechanistic
Models. Compr. Rev. Food Sci. Food Saf..

[32] Zhang Q., Heuchel M., Thüneman A. F., Machatschek R. (2025). The Role of
Diffusion in the Hydrolytic Degradation of Poly­(Lactic-Co-Glycolic
Acid): A Molecular Perspective. Polym. Degrad.
Stab..

[33] Baij L., Hermans J. J., Keune K., Iedema P. D. (2018). Time-Dependent ATR-FTIR
Spectroscopic Studies on Solvent Diffusion and Film Swelling in Oil
Paint Model Systems. Macromolecules.

[34] Dumitru A. C., Espinosa F. M., Garcia R., Foschi G., Tortorella S., Valle F., Dallavalle M., Zerbetto F., Biscarini F. (2015). In Situ Nanomechanical
Characterization of the Early Stages of Swelling and Degradation of
a Biodegradable Polymer. Nanoscale.

[35] Atiwesh G., Mikhael A., Parrish C. C., Banoub J., Le T.-A. T. (2021). Environmental
Impact of Bioplastic Use: A Review. Heliyon.

[36] Mori R. (2023). Replacing
All Petroleum-Based Chemical Products with Natural Biomass-Based Chemical
Products: A Tutorial Review. RSC Sustainability.

[37] Romão S., Bettencourt A., Ribeiro I. A. C. (2022). Novel Features of Cellulose-Based
Films as Sustainable Alternatives for Food Packaging. Polymers.

[38] Farha, O. K. ; Xin, J. ; Ma, K. ; Cheung, Y. H. Biosynthesis of Hierarchical Metal Organic Framework–Bacterial Cellulose Composites US 20,250,075,238 A1 2025

[39] Jahangiri F., Mohanty A. K., Misra M. (2024). Sustainable Biodegradable Coatings
for Food Packaging: Challenges and Opportunities. Green Chem..

[40] Wang J., Euring M., Ostendorf K., Zhang K. (2022). Biobased Materials
for Food Packaging. J. Bioresour. Bioprod..

[41] Tofanica B.-M., Mikhailidi A., Samuil C., Ungureanu O. C., Fortună M. E., Ungureanu E. (2024). Advances in Cellulose-Based Hydrogels:
Current Trends and Challenges. Gels.

[42] Karmakar A., Titi H. M., Goldberg I. (2011). Coordination
Polymers of 5-(2-Amino/Acetamido-4-Carboxyphenoxy)-Benzene-1,3-Dioic
Acids with Transition Metal Ions: Synthesis, Structure, and Catalytic
Activity. Cryst. Growth Des..

[43] Addison A. W., Rao T. N., Reedijk J., van Rijn J., Verschoor G. C. (1984). Synthesis
Structure, and Spectroscopic Properties of Copper­(II) Compounds Containing
Nitrogen–Sulphur Donor Ligands; the Crystal and Molecular Structure
of Aqua­[1,7-bis­(N-methylbenzimidazol-2′-yl)-2,6-dithiaheptane]­copper­(II). J. Chem. Soc., Dalton Trans..

[44] Costa I. F. M., Franco C. H. J., Nesterov D. S., André V., Pereira L. C. J., Kirillov A. M. (2024). Alkoxy-Bridged Dicopper
(II) Cores
Meet Tetracyanonickelate Linkers: Structural, Magnetic, and Theoretical
Investigation of Cu/Ni Coordination Polymers. J. Phys. Chem. C.

[45] Whitcomb D. R., Rajeswaran M. (2006). Designing
Silver Carboxylate Polymers: Crystal Structures
of Silver-Acetyl-Benzoate and Silver-1,2-Benzenedicarboxylate Monomethyl
Ester. Polyhedron.

[46] Blackman A. G., Schenk E. B., Jelley R. E., Krenske E. H., Gahan L. R. (2020). Five-Coordinate
Transition Metal Complexes and the Value of τ 5: Observations
and Caveats. Dalt. Trans..

[47] Diniz L. F., Franco C. H. J., Silva D. F., Martins L. S., Carvalho P. S., Souza M. A. C., Reis N. F. A., Fernandes C., Diniz R. (2021). Multicomponent Ionic
Crystals of Diltiazem with Dicarboxylic Acids
toward Understanding the Structural Aspects Driving the Drug-Release. Int. J. Pharm..

[48] Kyratzis N., Cao W., Izgorodina E. I., Turner D. R. (2018). Structural Changes
in Coordination Polymers in Response to Small Changes in Steric Bulk
(H vs. Me): An Experimental and Theoretical Study. CrystEngComm.

[49] de
Azevedo-França J. A., Barrias E., Franco C. H. J., Villarreal W., Vieira E. G., Ferreira A. M. D. C., de Souza W., Navarro M. (2022). Promising Fluconazole Based Zinc­(II)
and Copper­(II) Coordination Polymers against Chagas Disease. J. Inorg. Biochem..

[50] Brea R. J., Hernández A., Criado A., Mosquera J. (2024). Deciphering the Concept
of Solubility by Strategically Using the Counterion Effect in Charged
Molecules. J. Chem. Educ..

[51] Muniz F. T. L., Miranda M. A. R., Morilla
dos Santos C., Sasaki J. M. (2016). The Scherrer Equation and the Dynamical
Theory of X-Ray
Diffraction. Acta Crystallogr., Sect. A: Found.
Adv..

[52] Mal S., Franco C. H. J., Kumar B., Kirillov A. M., Das S. (2025). Exploring
Uracil Derivatives: Synthesis, Crystal Structure Insights, and Antibacterial
Activity. CrystEngComm.

[53] Agarwal S. (2021). Major Factors
Affecting the Characteristics of Starch Based Biopolymer Films. Eur. Polym. J..

[54] Liscano Y., Salamanca C. H., Vargas L., Cantor S., Laverde-Rojas V., Oñate-Garzón J. (2019). Increases in Hydrophilicity and Charge
on the Polar Face of Alyteserin 1c Helix Change Its Selectivity towards
Gram-Positive Bacteria. Antibiotics.

[55] Pearce A. K., O’Reilly R. K. (2021). Polymers
for Biomedical Applications: The Importance
of Hydrophobicity in Directing Biological Interactions and Application
Efficacy. Biomacromolecules.

[56] Draviana H. T., Fitriannisa I., Khafid M., Krisnawati D. I., Widodo, Lai C.-H., Fan Y.-J., Kuo T.-R. (2023). Size and Charge Effects of Metal
Nanoclusters on Antibacterial Mechanisms. J.
Nanobiotechnol..

[57] Mirnejad R., Fasihi-Ramandi M., Behmard E., Najafi A., Moosazadeh
Moghaddam M. (2023). Interaction of Antibacterial CM11 Peptide with the
Gram-Positive and Gram-Negative Bacterial Membrane Models: A Molecular
Dynamics Simulations Study. Chem. Pap..

[58] Tasleem M., Matouk A. M., Abbas M. (2025). Design of Short Peptides for the
Reduction of Silver Ions and Stabilization of Nanocomposites in Combating
Bacterial Infections. ChemBioChem.

[59] Fernandes T. A., Costa I. F. M., Jorge P., Sousa A. C., André V., Cabral R. G., Cerca N., Kirillov A. M. (2022). Hybrid Silver­(I)-Doped
Soybean Oil and Potato Starch Biopolymer Films to Combat Bacterial
Biofilms. ACS Appl. Mater. Interfaces.

[60] Kropp G. A., McMillian C. N., Mase J. D., Kandagiri S., Nowak E. S., Schulz M. D. (2026). Recent
Developments in Antimicrobial
Polymers for Biofilm Inhibition. Chem. Commun..

[61] Luo T., Yao T., Zhan Z., Gan B., Wu X., Xu H. (2026). Recent Progress
on Silver-Based Antibacterial Food Packaging. Compr. Rev. Food Sci. Food Saf..

[62] Subramani T., Vuppu S. (2026). Targeting Super Bugs:
Metal Complexes as Emerging Anti-Microbial
and Anti-Biofilm Agents. Arch. Microbiol..

