# Supplementary material for: Silver Sulfobenzoate Coordination Polymers as Bioactive Dopants in Antibacterial and Antibiofilm Cellulose Films
Source: ACS Appl Mater Interfaces. 2026 Jun 4;18(23):32217–30. doi: 10.1021/acsami.6c00401 (PMC13288408; doi:10.1021/acsami.6c00401)
Supplement: Supplementary file 1 [file am6c00401_si_001.pdf]

Supporting Information (SI)

Silver Sulfobenzoate Coordination Polymers as Bioactive Dopants in Antibacterial and Antibiofilm Cellulose Films

Rafaela G. Cabral,[#,§] Tiago A. Fernandes,[#,‡] Chris H. J. Franco,[#,†] Paula Jorge,[±,¶] Ivo M. F. Bragança,[√,□] Ana Catarina Sousa,[#,§] Nuno Cerca[*,±¶] and Alexander M. Kirillov[*,#]

[#]MINDlab: Molecular Design & Innovation Laboratory, Centro de Química Estrutural, Institute of Molecular Sciences, Instituto Superior Técnico, Universidade de Lisboa, Av. Rovisco Pais, 1049-001, Lisboa, Portugal. https://MINDlab.pt e-mail: kirillov@tecnico.ulisboa.pt

[§]Departamento de Engenharia Química, ISEL – Instituto Superior de Engenharia de Lisboa, Instituto Politécnico de Lisboa, R. Conselheiro Emídio Navarro, 1, 1959-007 Lisboa, Portugal.

[‡]Departamento de Ciências e Tecnologia (DCeT), Universidade Aberta, 1000-013 Lisboa, Portugal.

[†]Institute of Low Temperature and Structure Research, Polish Academy of Sciences, Okólna 2, 50-422 Wroclaw, Poland.

[±]Centre of Biological Engineering, University of Minho, Campus de Gualtar, 4710-057 Braga, Portugal. e-mail: nunocerca@ceb.uminho.pt

[¶]LABBELS−Associate Laboratory, University of Minho, 4710-057 Braga/Guimarães, Portugal.

[√]IDMEC, Instituto Superior Técnico, Universidade de Lisboa, Portugal.

[□]CIMOSM, Instituto Superior de Engenharia de Lisboa, Instituto Politécnico de Lisboa, Portugal.

Supporting Information contains: materials and methods, FTIR-ATR spectra (Fig. S1−S4); swelling ability of $[EC]_n$ and $[CA]_n$ biopolymers (Fig. S5); TGA data (Figs. S6-S9); crystal data and structure refinement for **CP1** and **CP2** (Table S1); additional structural details (Tables S3-S6) and figures (Figs S10-S13) for **CP1** and **CP2**; PXRD patterns (Figs. S14-16); SEM-EDS data (Figs. S17-S22, S30); mechanical analysis details and properties (Tables S7-S8); additional antimicrobial data (Figs. S23−S28), and UV-vis spectra (Fig. S29); crystallographic data in CIF format (CCDC Numbers 2502323-2502324). (PDF).

Materials and Methods. All chemicals and solvents were purchased from commercial sources. FTIR-ATR spectra were recorded on a Shimadzu IRAffinity-1S apparatus equipped with an ATR ZnSe Performance Crystal Plate accessory. Absorbance spectra were collected in the 4000–650 cm^{-1} range with a 2.0 cm^{-1} resolution using 64 co-added scans (abbreviations: vs − very strong, s − strong, m – medium, w – weak, br – broad, sh − shoulder). Elemental analyses were run on a Perkin Elmer PE 2400 Series II analyzer by Laboratory of Analyses of IST. SEM and SEM-EDX were acquired using a Thermo Scientific, Phenom ProX G6 Desktop SEM, with the accelerating voltage of 25.0 kV. For silver release studies, the determination of Ag^{+} content (after storing the biopolymer film samples in PBS solutions for 48h) was performed by ICP-OES (Perkin Elmer Optical Emission Spectrometer Optima 2000 DV) with the following operation conditions: RF power 1300 W, auxiliary gas flow 0.2 L min^{-1} nebulizer gas flow 0.6 L min^{-1}, plasma flow 15 L min^{-1}, Sample Flow Rate 1.50 mL min^{-1}, and Cu analytical line 327.393 nm. These analyses were performed by a certified laboratory (Laboratory of Analyses, IST, Portugal). Thermogravimetric analyses (TGA) were carried out on a Metler Toledo TGA/DSC-1/1600 HF in the temperature range between 30 and 800 °C and at a heating rate of 10 °C min^{-1} under air. TS and elongation tests were conducted with an Instron 5966 equipment, using film samples with dimensions of approximately 60 mm × 12 mm × 0.04 mm.

X-ray Crystallography

Single-crystal X-ray diffraction data for compounds **CP1** and **CP2** were collected at room temperature using a Bruker AXS APEXIII diffractometer equipped with a PHOTON II CCD detector and employing Mo-Kα radiation (λ = 0.71073 Å). Data collection and reduction for **CP1** were carried out using the APEXIII and SAINT software packages, with absorption corrections applied using SADABS. For **CP2**, data reduction and absorption correction were performed using the CrysAlisPro software package (version 1.171.40.69a, 2020). Structure solution for both compounds was achieved using SHELXT via intrinsic phasing, and refinements were performed using SHELXL with full-matrix least-squares minimization on F^2, all within the Olex2 graphical interface. All non-hydrogen atoms were refined anisotropically. Hydrogen atoms were placed in geometrically calculated positions and refined using a riding model. C–H bond distances were constrained to 0. Å for aromatic hydrogen atoms and 0.96 Å for methyl groups, with isotropic displacement parameters set at $U_{iso}(H) = 1.2U_{eq}(C)$ in both compounds. For **CP2**, residual electron density indicative of disordered solvent was observed in the asymmetric unit. A solvent mask was applied using the MASK routine in Olex2, which identified a total of 260 electrons in a void of 378 $Å^3$, corresponding to one solvent-accessible region per unit cell. This electron count is consistent with the presence of approximately three water molecules per asymmetric unit, totaling 240 electrons per unit cell. Due to their high degree of disorder and lack of defined atomic positions, these water molecules were not modeled explicitly but their contribution was considered in the refinement process. CCDC 2502323-2502324.

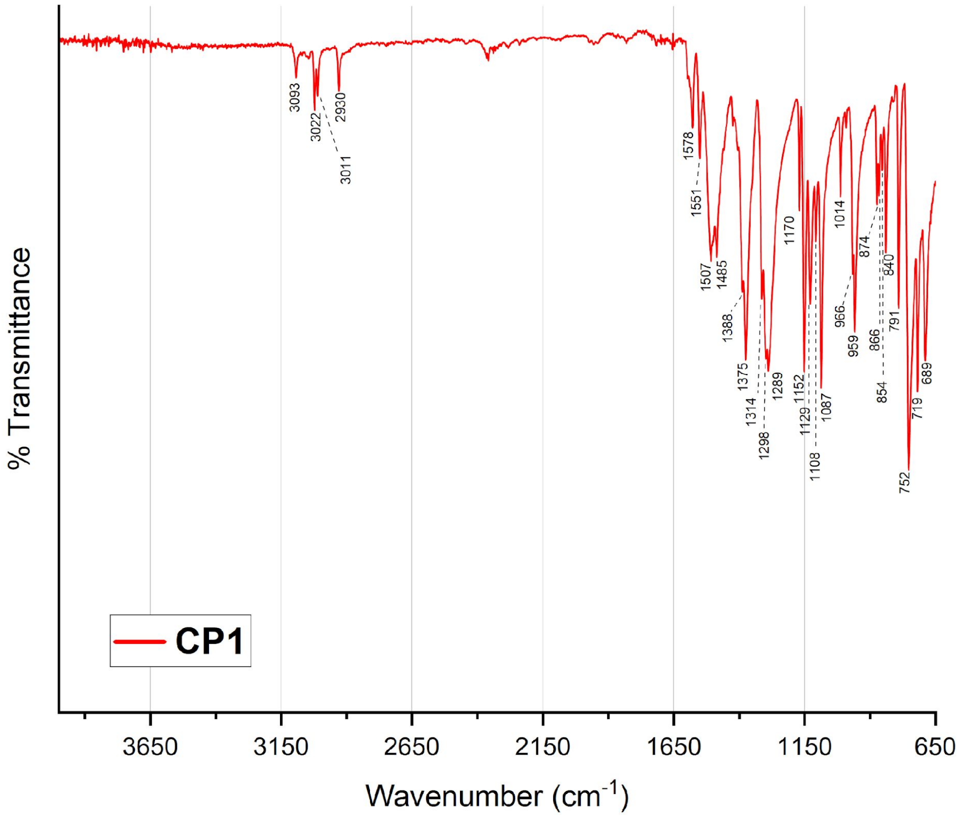

Figure S1. FTIR-ATR spectrum of **CP1**.

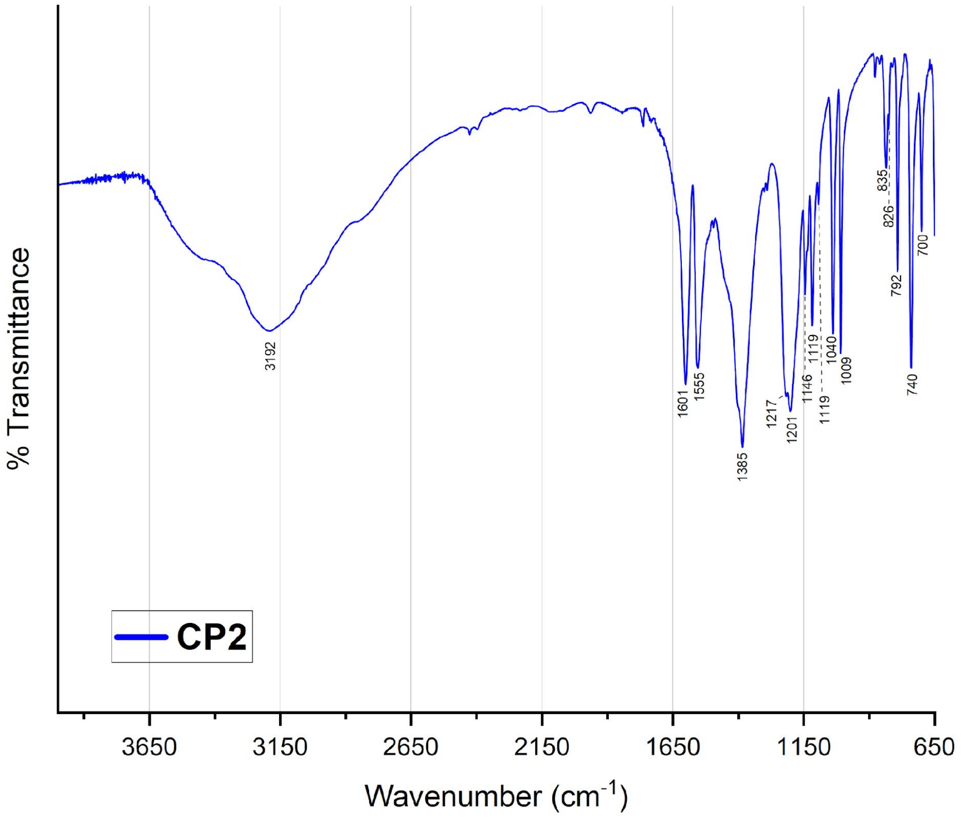

Figure S2. FTIR-ATR spectrum of **CP2**.

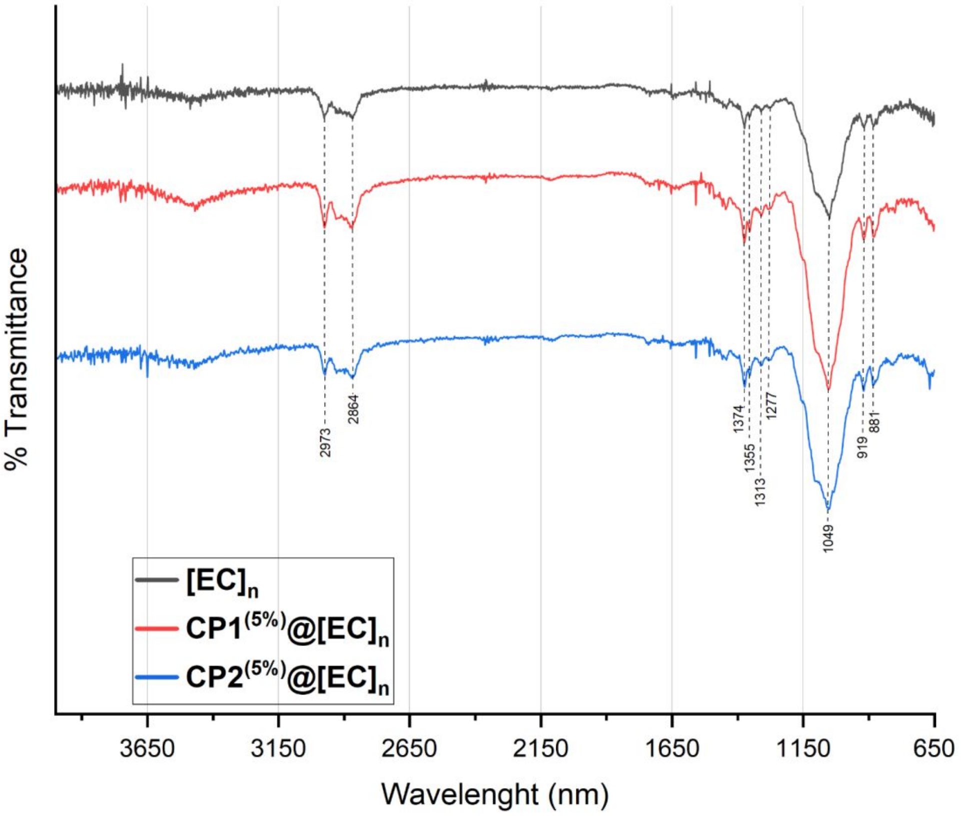

Figure S3. FTIR-ATR spectra of $[EC]_n$, **CP1**$^{(5\%)}$@$[EC]_n$, and **CP2**$^{(5\%)}$@$[EC]_n$.

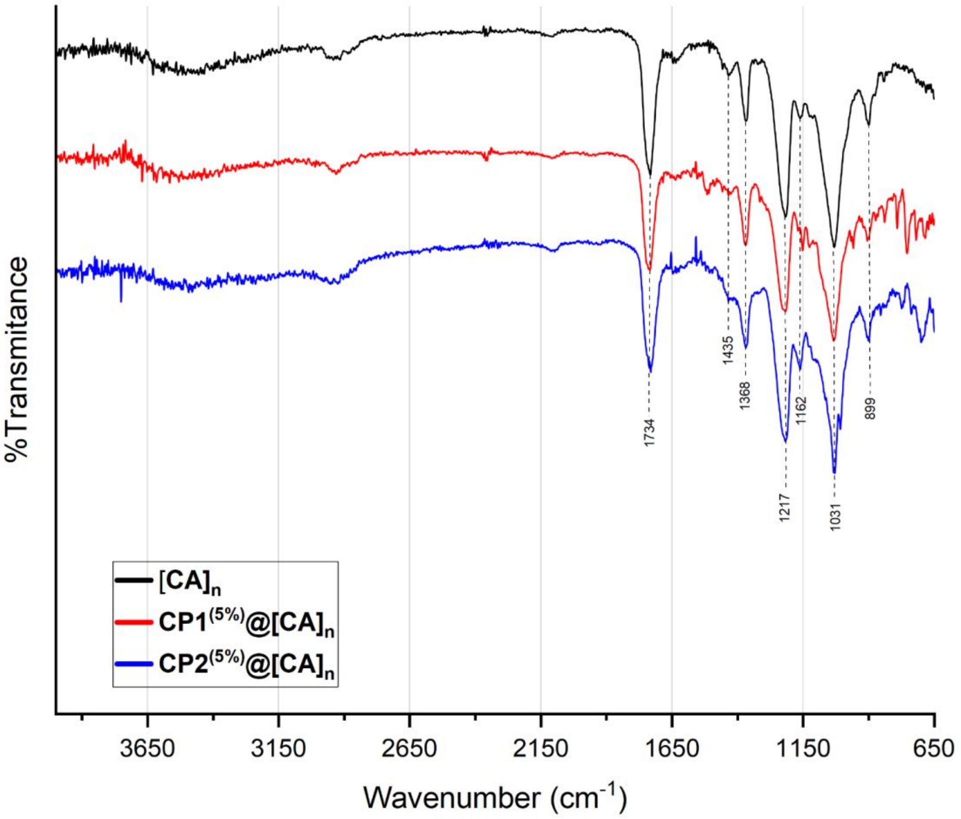

Figure S4. FTIR-ATR spectra of $[CA]_n$, **CP1**$^{(5\%)}$@$[CA]_n$, and **CP2**$^{(5\%)}$@$[CA]_n$.

Swelling Tests

The swelling behavior of the produced biopolymer films was evaluated at physiological conditions, in PBS buffer solution at pH 7.4 at 37 °C for 24 h. Dried samples (10 mm × 10 mm) were weighed and immersed in containers with 10 mL to swell to equilibrium. After 24 h, the samples were removed, softly wiped with absorbent paper, and weighed. The swelling ratio (%) was calculated according to the following expression:

$$Swelling\ ratio = \frac{w_f - w_i}{w_i} \times 100\% \qquad (eq.\ 1)$$

where w_i and w_f are the weight of the samples before and after the swelling, respectively. Studies were carried out in triplicate.

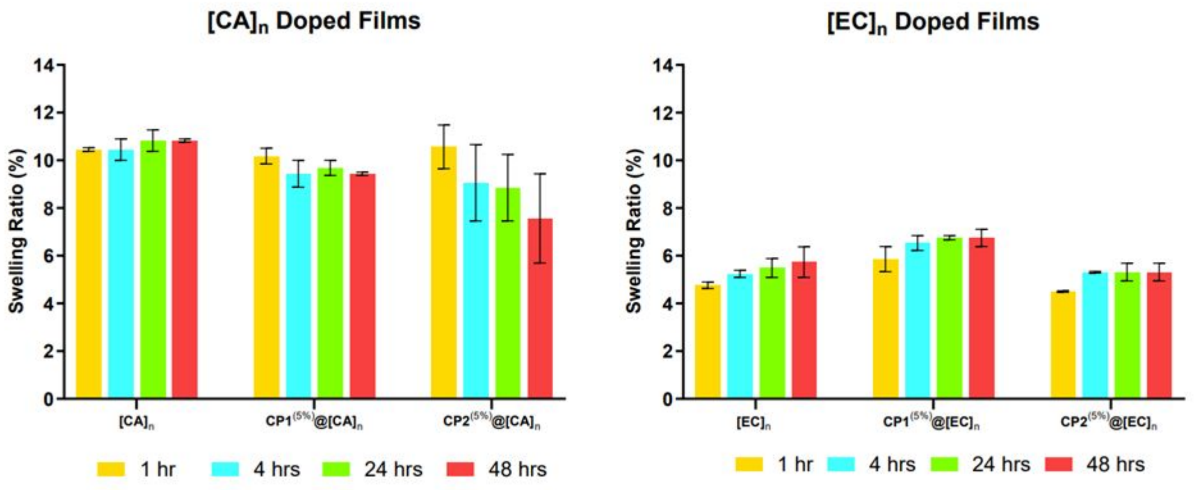

Figure S5. Swelling ability of $[CA]_n$, **CP1**$^{(5\%)}$@$[CA]_n$, **CP2**$^{(5\%)}$@$[CA]_n$, $[EC]_n$, **CP1**$^{(5\%)}$@$[EC]_n$ and **CP2**$^{(5\%)}$@$[CA]_n$ films at 1-48 h.

Thermogravimetric Analysis

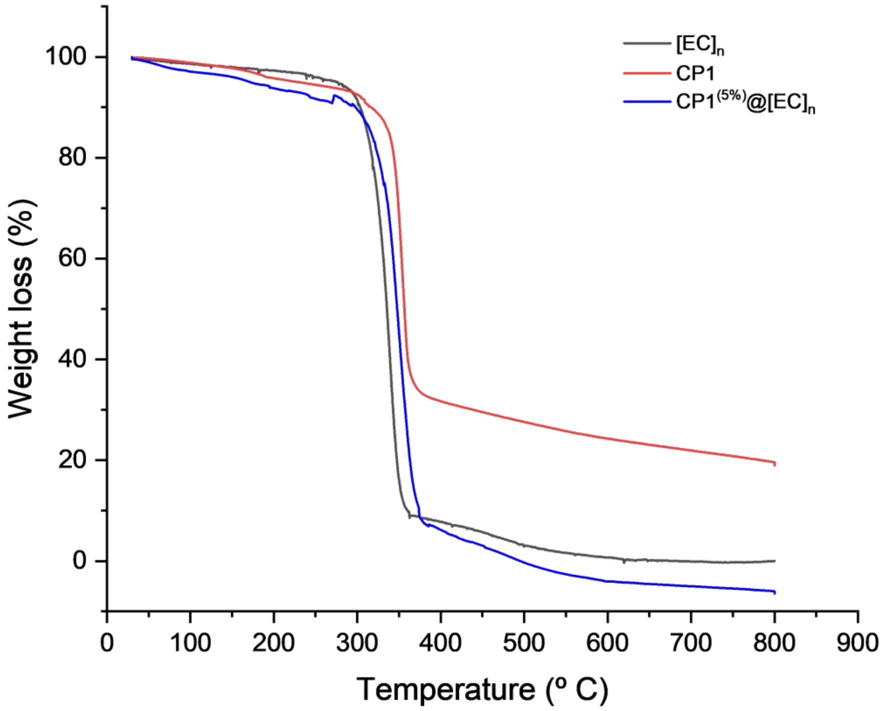

Figure S6. TGA plots of $[EC]_n$, **CP1**, and **CP1**$^{(5\%)}$@$[EC]_n$.

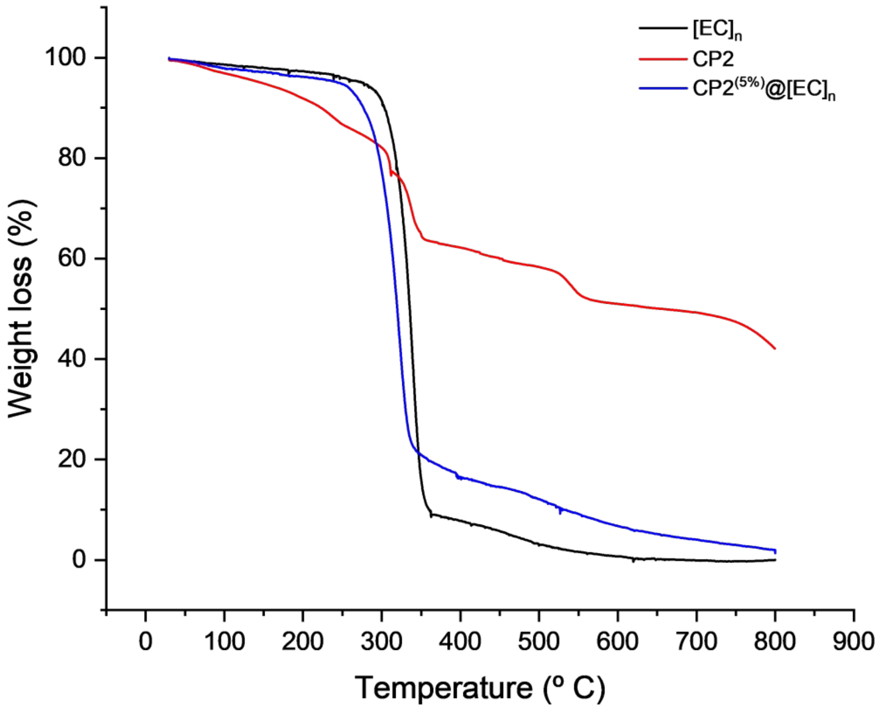

Figure S7. TGA plots of $[EC]_n$, **CP2**, and **CP2**$^{(5\%)}$@$[EC]_n$.

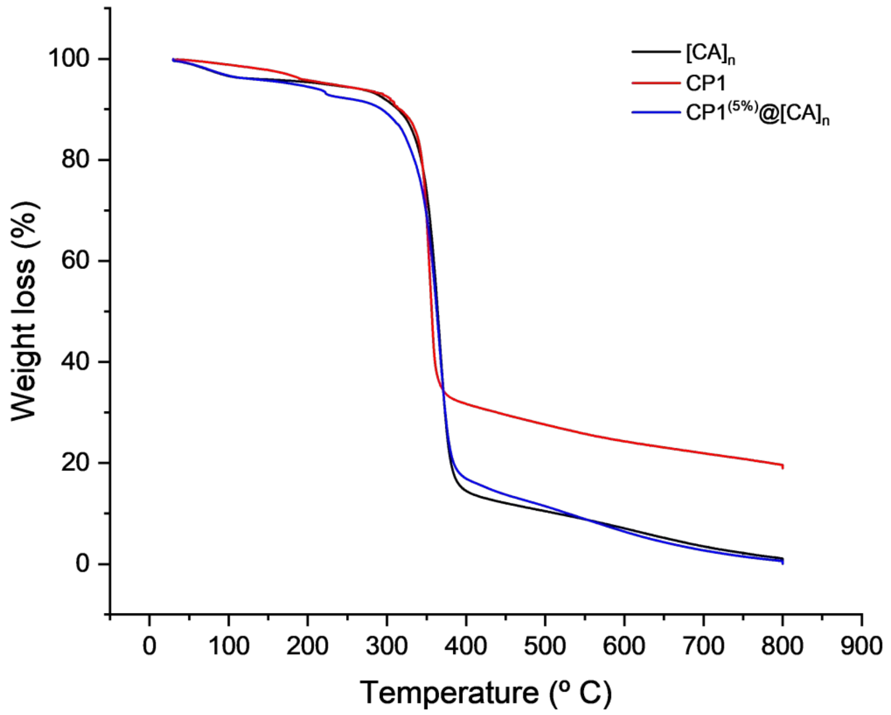

Figure S8. TGA plots of $[CA]_n$, **CP1**, and **CP1**$^{(5\%)}$@$[CA]_n$.

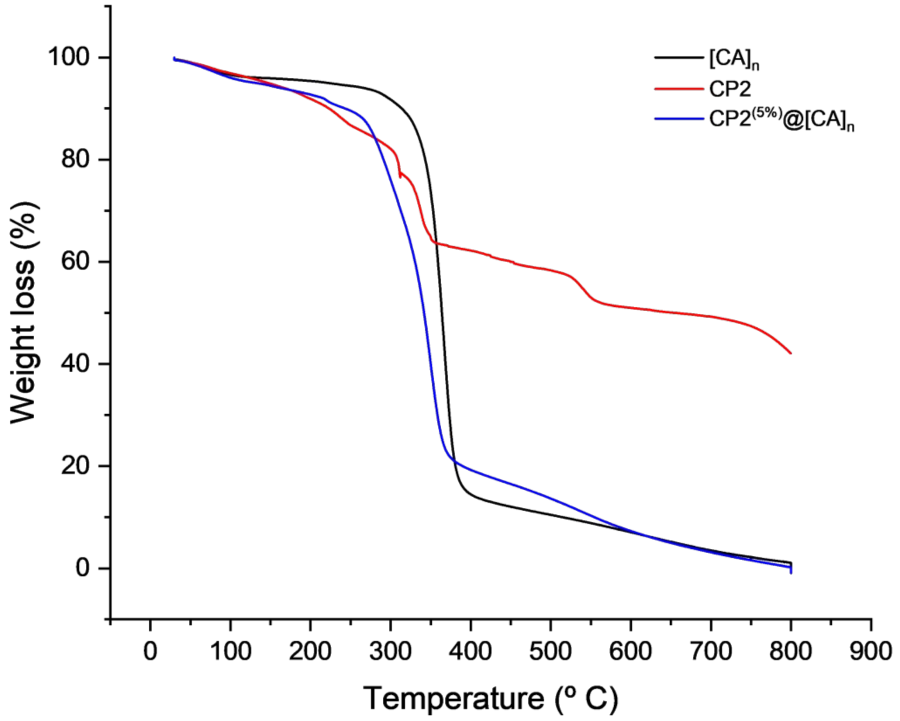

Figure S9. TGA plots of $[CA]_n$, **CP2**, and **CP2**$^{(5\%)}$@$[CA]_n$.

Table S1. Crystal data and structure refinement for **CP1** and **CP2**.

	**CP1**	**CP2**
Empirical formula	$C_8H_7AgO_4S$	$C_7H_{10}AgKO_9S$
Formula weight	307.07	417.18
Temperature/K	298	293(2)
Crystal system	monoclinic	monoclinic
Space group	$P2_1/c$	C2/m
a/Å	15.2774(15)	5.7026(2)
b/Å	9.3452(9)	19.8295(9)
c/Å	6.3690(6)	20.2731(5)
α/°	90	90
β/°	100.982(3)	92.903(3)
γ/°	90	90
Volume/Å^3	892.65(15)	2289.53(14)
Z	4	8
ρ_{calc}g/cm^3	2.285	2.421
μ/mm^{-1}	2.473	2.349
Crystal size/mm^3	0.095 × 0.073 × 0.051	0.091 × 0.03 × 0.025
Radiation	MoKα (λ = 0.71073)	Mo Kα (λ = 0.71073)
Reflections collected	12846	33855
Independent reflections	1819 [R_{int} = 0.0362, R_{sigma} = 0.0237]	2160 [R_{int} = 0.0602, R_{sigma} = 0.0231]
Data/restraints/parameters	1819/0/128	2160/0/154
Goodness-of-fit on F^2	1.086	1.146
Final R indexes [I>=2σ (I)]	R_1 = 0.0210, wR_2 = 0.0547	R_1 = 0.0767, wR_2 = 0.1886
Largest diff. peak/hole / e Å^{-3}	0.39/-0.50	1.88/-1.76

Table S2. Bond Lengths for **CP1**.

Atom	Atom	Length/Å	Atom	Atom	Length/Å
Ag1	Ag1^1	2.9121(4)	O1	C1	1.250(3)
Ag1	O2^1	2.1906(17)	C2	C3	1.393(3)
Ag1	O2^2	2.5692(16)	C2	C1	1.509(3)
Ag1	O1	2.2035(16)	C2	C7	1.393(3)
S1	O3	1.435(2)	C5	C6	1.390(3)
S1	O4	1.440(2)	C5	C4	1.389(3)
S1	C5	1.770(2)	C6	C7	1.377(3)
S1	C8	1.747(3)	C3	C4	1.382(3)
O2	C1	1.272(3)			

^{1}1-x,1-y,-z; ^{2}1-x,-1/2+y,1/2-z

Table S3. Bond Angles for **CP1**.

Atom	Atom	Atom	Angle/˚	Atom	Atom	Atom	Angle/˚
O2^1	Ag1	Ag1^2	168.59(4)	C1	O1	Ag1	126.78(15)
O2^2	Ag1	Ag1^2	82.09(4)	C3	C2	C1	120.47(19)
O2^2	Ag1	O2^1	109.31(5)	C3	C2	C7	119.3(2)
O2^2	Ag1	O1	158.57(7)	C7	C2	C1	120.10(19)
O1	Ag1	Ag1^2	79.45(4)	C6	C5	S1	119.53(17)
O1	Ag1	O2^1	89.45(6)	C4	C5	S1	119.66(17)
O3	S1	O4	118.47(15)	C4	C5	C6	120.8(2)
O3	S1	C5	108.37(12)	C7	C6	C5	119.4(2)
O3	S1	C8	108.34(14)	C4	C3	C2	120.6(2)
O4	S1	C5	108.03(12)	O2	C1	C2	116.93(19)
O4	S1	C8	108.11(14)	O1	C1	O2	125.8(2)
C8	S1	C5	104.67(12)	O1	C1	C2	117.21(19)
Ag1^2	O2	Ag1^3	106.29(6)	C6	C7	C2	120.6(2)
C1	O2	Ag1^2	122.45(14)	C3	C4	C5	119.2(2)
C1	O2	Ag1^3	120.23(14)				

^{1}1-x,-1/2+y,1/2-z; ^{2}1-x,1-y,-z; ^{3}1-x,1/2+y,1/2-z

Table S4. Bond Lengths for **CP2**.

Atom	Atom	Length/Å	Atom	Atom	Length/Å
Ag1	Ag1[1]	2.8274(16)	O2	C1	1.231(11)
Ag1	Ag1[2]	3.200(2)	O3	K1	2.761(8)
Ag1	O1[3]	2.471(7)	O3	K1[5]	3.004(8)
Ag1	O1[1]	2.235(6)	C5	C6	1.383(11)
Ag1	O2	2.180(7)	C5	C4	1.393(12)
S1	O4	1.448(7)	C6	C7	1.371(11)
S1	O5	1.433(7)	C2	C7	1.389(12)
S1	O3	1.437(8)	C2	C3	1.374(13)
S1	C5	1.779(7)	C2	C1	1.498(11)
S1	K1[4]	3.748(2)	C4	C3	1.404(11)
S1	K1[5]	3.611(2)	K2	O6[3]	2.95(8)
S1	K2	3.4630(19)	K2	O6[6]	2.82(9)
O4	K1[4]	2.825(8)	K2	O6	2.82(9)
O4	K2	2.734(7)	K2	O6[7]	2.95(9)
O4	K3[3]	2.853(10)	K3	O6[7]	2.99(3)
O5	K1[5]	3.192(8)	K3	O6	2.12(4)
O5	K2	3.141(8)	K3	O7	1.62(5)
O5	K3	2.648(10)	O6	O6[7]	0.90(5)
O1	C1	1.249(11)			

[1]2-x,+y,-z; [2]5/2-x,1/2-y,-z; [3]1+x,+y,+z; [4]3/2-x,1/2-y,1-z; [5]1/2-x,1/2-y,1-z; [6]1-x,1-y,1-z; [7]-x,1-y,1-z

Table S5. Bond Angles for **CP2**.

Atom	Atom	Atom	Angle/˚	Atom	Atom	Atom	Angle/˚
Ag1^{1}	Ag1	Ag1^{2}	84.26(3)	O4	K2	O4^{10}	98.8(3)
O1^{3}	Ag1	Ag1^{1}	165.40(16)	O4^{11}	K2	O4^{10}	81.2(3)
O1^{1}	Ag1	Ag1^{1}	84.76(17)	O4	K2	O4^{11}	180.0
O1^{1}	Ag1	Ag1^{2}	73.4(2)	O4^{10}	K2	O4^{9}	180.0(2)
O1^{3}	Ag1	Ag1^{2}	97.7(2)	O4	K2	O4^{9}	81.2(3)
O1^{1}	Ag1	O1^{3}	82.0(2)	O4^{11}	K2	O4^{9}	98.8(3)
O2	Ag1	Ag1^{2}	105.8(3)	O4	K2	O5^{11}	132.63(19)
O2	Ag1	Ag1^{1}	76.1(2)	O4	K2	O5^{9}	75.8(2)
O2	Ag1	O1^{1}	160.8(3)	O4^{10}	K2	O5^{9}	132.63(19)
O2	Ag1	O1^{3}	116.9(3)	O4^{11}	K2	O5^{9}	104.2(2)
O4	S1	C5	104.8(4)	O4^{10}	K2	O5^{10}	47.37(19)
O4	S1	K1^{4}	41.3(3)	O4^{11}	K2	O5	132.63(19)
O4	S1	K1^{5}	115.2(3)	O4^{9}	K2	O5	75.8(2)
O4	S1	K2	48.8(3)	O4	K2	O5^{10}	104.2(2)
O5	S1	O4	112.0(5)	O4^{9}	K2	O5^{11}	104.2(2)
O5	S1	O3	112.5(5)	O4^{10}	K2	O5	104.2(2)
O5	S1	C5	108.5(4)	O4^{9}	K2	O5^{10}	132.63(19)
O5	S1	K1^{5}	61.7(3)	O4^{11}	K2	O5^{11}	47.37(19)
O5	S1	K1^{4}	142.4(3)	O4^{10}	K2	O5^{11}	75.8(2)
O5	S1	K2	65.1(3)	O4^{11}	K2	O5^{10}	75.8(2)
O3	S1	O4	111.8(5)	O4	K2	O5	47.37(19)
O3	S1	C5	106.7(4)	O4^{9}	K2	O5^{9}	47.37(19)
O3	S1	K1^{5}	54.1(3)	O4^{10}	K2	O6^{11}	73.6(4)
O3	S1	K1^{4}	72.6(4)	O4^{9}	K2	O6^{12}	63.4(4)
O3	S1	K2	119.7(3)	O4^{10}	K2	O6^{12}	116.6(4)
C5	S1	K1^{5}	139.7(3)	O4^{11}	K2	O6	73.6(4)
C5	S1	K1^{4}	105.0(3)	O4^{11}	K2	O6^{3}	116.6(4)
C5	S1	K2	132.2(3)	O4^{9}	K2	O6^{3}	116.6(4)
K1^{5}	S1	K1^{4}	101.59(6)	O4^{10}	K2	O6^{3}	63.4(4)
S1	O4	K1^{4}	119.0(4)	O4^{11}	K2	O6^{11}	106.4(4)
S1	O4	K2	107.8(4)	O4^{10}	K2	O6	106.4(4)
S1	O4	K3^{3}	125.9(5)	O4	K2	O6^{11}	73.6(4)
K1^{4}	O4	K3^{3}	103.4(3)	O4	K2	O6	106.4(4)
S1	O5	K1^{5}	95.0(4)	O4^{9}	K2	O6	73.6(4)
S1	O5	K2	90.5(4)	O4^{9}	K2	O6^{11}	106.4(4)
S1	O5	K3	165.7(6)	O4	K2	O6^{3}	63.4(4)
K3	O5	K1^{5}	99.1(3)	O4^{11}	K2	O6^{12}	63.4(4)
K3	O5	K2	86.4(3)	O4	K2	O6^{12}	116.6(4)
Ag1^{1}	O1	Ag1^{6}	95.5(2)	O5^{10}	K2	O5^{9}	180.00(18)
C1	O1	Ag1^{6}	137.7(6)	O5^{9}	K2	O5	105.2(3)
C1	O1	Ag1^{1}	117.6(6)	O5^{11}	K2	O5	180.0
C1	O2	Ag1	130.3(6)	O5^{10}	K2	O5	74.8(3)
S1	O3	K1	158.1(5)	O5^{11}	K2	O5^{9}	74.8(3)
S1	O3	K1^{5}	103.1(4)	O5^{11}	K2	O5^{10}	105.2(3)
K1	O3	K1^{5}	84.10(19)	O6^{11}	K2	O5^{10}	120.4(4)
C6	C5	S1	119.2(6)	O6^{11}	K2	O5^{11}	59.6(4)
C6	C5	C4	120.1(7)	O6^{12}	K2	O5^{9}	107.1(6)
C4	C5	S1	120.7(6)	O6^{3}	K2	O5	107.1(6)
C7	C6	C5	120.3(7)	O6	K2	O5^{10}	59.6(4)
C7	C2	C1	118.8(8)	O6^{12}	K2	O5^{11}	107.1(6)
C3	C2	C7	119.6(7)	O6^{12}	K2	O5^{10}	72.9(6)
C3	C2	C1	121.6(8)	O6	K2	O5	59.6(4)
C6	C7	C2	120.6(8)	O6^{3}	K2	O5^{11}	72.9(6)
C5	C4	C3	119.0(8)	O6	K2	O5^{9}	120.4(4)
C2	C3	C4	120.5(8)	O6^{12}	K2	O5	72.9(6)
O1	C1	C2	119.4(8)	O6	K2	O5^{11}	120.4(4)
O2	C1	O1	124.1(8)	O6^{11}	K2	O5^{9}	59.6(4)
O2	C1	C2	116.5(8)	O6^{11}	K2	O5	120.4(4)
S1^{7}	K1	S1^{4}	70.88(5)	O6^{3}	K2	O5^{9}	72.9(6)
S1^{8}	K1	S1^{4}	151.40(11)	O6^{3}	K2	O5^{10}	107.1(6)
S1^{7}	K1	S1^{8}	101.59(6)	O6^{11}	K2	O6^{12}	162.2(12)
S1^{5}	K1	S1^{8}	70.88(5)	O6	K2	O6^{3}	162.2(12)
S1^{5}	K1	S1^{4}	101.59(6)	O6^{12}	K2	O6^{3}	180.0
S1^{5}	K1	S1^{7}	150.29(12)	O6^{11}	K2	O6^{3}	17.8(12)
O4^{8}	K1	S1^{8}	19.76(15)	O6	K2	O6^{12}	17.8(12)
O4^{4}	K1	S1^{5}	92.78(15)	O6^{11}	K2	O6	180.0
O4^{4}	K1	S1^{4}	19.76(15)	O4^{6}	K3	O4^{13}	93.4(4)

Atom	Atom	Atom	Angle/°	Atom	Atom	Atom	Angle/°
O4^{8}	K1	S1^{5}	70.45(14)	O4^{13}	K3	K1^{5}	113.9(3)
O4^{4}	K1	S1^{8}	131.71(18)	O4^{6}	K3	K1^{5}	38.07(18)
O4^{8}	K1	S1^{4}	131.71(18)	O4^{13}	K3	K1^{14}	38.07(18)
O4^{4}	K1	S1^{7}	70.45(14)	O4^{6}	K3	K1^{14}	113.9(3)
O4^{8}	K1	S1^{7}	92.78(15)	O4^{13}	K3	K2	101.3(3)
O4^{8}	K1	O4^{4}	112.2(3)	O4^{6}	K3	K2^{6}	47.5(2)
O4^{4}	K1	O5^{5}	70.71(19)	O4^{6}	K3	K2	101.3(3)
O4^{8}	K1	O5^{5}	73.8(2)	O4^{13}	K3	K2^{6}	47.5(2)
O4^{4}	K1	O5^{7}	73.8(2)	O4^{6}	K3	O6^{12}	69.3(11)
O4^{8}	K1	O5^{7}	70.71(19)	O4^{13}	K3	O6^{12}	69.3(11)
O4^{8}	K1	O3^{5}	78.7(2)	O5^{10}	K3	O4^{13}	78.8(3)
O4^{4}	K1	O3^{7}	78.7(2)	O5	K3	O4^{6}	78.8(3)
O4^{8}	K1	O3^{7}	109.8(2)	O5	K3	O4^{13}	148.7(5)
O4^{4}	K1	O3^{5}	109.8(2)	O5^{10}	K3	O4^{6}	148.7(5)
O5^{7}	K1	S1^{8}	82.63(14)	O5	K3	O5^{10}	92.2(5)
O5^{5}	K1	S1^{5}	23.28(13)	O5^{10}	K3	K1^{14}	45.0(2)
O5^{5}	K1	S1^{7}	129.67(16)	O5	K3	K1^{14}	118.3(4)
O5^{7}	K1	S1^{5}	129.67(16)	O5^{10}	K3	K1^{5}	118.3(4)
O5^{5}	K1	S1^{4}	82.63(14)	O5	K3	K1^{5}	45.0(2)
O5^{7}	K1	S1^{4}	81.86(15)	O5^{10}	K3	K2^{6}	112.6(3)
O5^{5}	K1	S1^{8}	81.86(15)	O5	K3	K2^{6}	112.6(3)
O5^{7}	K1	S1^{7}	23.28(13)	O5^{10}	K3	K2	52.0(3)
O5^{7}	K1	O5^{5}	113.7(3)	O5	K3	K2	52.0(3)
O3^{5}	K1	S1^{5}	22.81(15)	O5^{10}	K3	O6^{12}	79.6(12)
O3^{9}	K1	S1^{4}	76.86(19)	O5	K3	O6^{12}	79.6(12)
O3^{5}	K1	S1^{4}	112.61(16)	K1^{14}	K3	K1^{5}	110.1(2)
O3^{9}	K1	S1^{8}	128.7(2)	K2^{6}	K3	K2	96.3(2)
O3	K1	S1^{8}	76.86(19)	O6	K3	O4^{6}	72.3(15)
O3^{7}	K1	S1^{4}	71.28(16)	O6	K3	O4^{13}	72.3(15)
O3	K1	S1^{5}	117.05(18)	O6	K3	O5^{10}	76.5(16)
O3^{9}	K1	S1^{5}	87.60(16)	O6	K3	O5	76.5(16)
O3^{7}	K1	S1^{5}	170.98(18)	O6^{12}	K3	K1^{5}	55.07(13)
O3^{5}	K1	S1^{8}	71.28(16)	O6	K3	K1^{5}	55.09(15)
O3	K1	S1^{4}	128.7(2)	O6^{12}	K3	K1^{14}	55.07(13)
O3^{7}	K1	S1^{8}	112.61(16)	O6	K3	K1^{14}	55.09(14)
O3^{5}	K1	S1^{7}	170.98(18)	O6	K3	K2	43(2)
O3^{7}	K1	S1^{7}	22.81(15)	O6	K3	K2^{6}	53(2)
O3^{9}	K1	S1^{7}	117.05(18)	O6^{12}	K3	K2	47.5(17)
O3	K1	S1^{7}	87.60(16)	O6^{12}	K3	K2^{6}	48.8(17)
O3^{9}	K1	O4^{8}	146.0(2)	O6	K3	O6^{12}	5(4)
O3^{9}	K1	O4^{4}	94.0(2)	O7	K3	O4^{6}	107.7(8)
O3	K1	O4^{8}	94.0(2)	O7	K3	O4^{13}	107.7(8)
O3	K1	O4^{4}	146.0(2)	O7	K3	O5^{10}	103.5(8)
O3^{5}	K1	O5^{7}	147.7(2)	O7	K3	O5	103.5(8)
O3	K1	O5^{7}	96.3(2)	O7	K3	K1^{14}	124.91(13)
O3^{5}	K1	O5^{5}	45.18(19)	O7	K3	K1^{5}	124.91(13)
O3^{7}	K1	O5^{5}	147.7(2)	O7	K3	K2^{6}	126.6(12)
O3	K1	O5^{5}	140.3(2)	O7	K3	K2	137.1(12)
O3^{9}	K1	O5^{5}	96.3(2)	O7	K3	O6^{12}	175(2)
O3^{7}	K1	O5^{7}	45.18(19)	O7	K3	O6	180(3)
O3^{9}	K1	O5^{7}	140.3(2)	K3	O6	K2	106(2)
O3	K1	O3^{5}	95.90(19)	K3	O6	K2^{6}	91(3)
O3^{7}	K1	O3^{5}	165.2(3)	K3	O6	K3^{12}	175(4)
O3^{9}	K1	O3^{5}	71.96(19)	O6^{12}	O6	K2	89(10)
O3^{9}	K1	O3^{7}	95.90(18)	O6^{12}	O6	K2^{6}	73(10)
O3	K1	O3^{9}	72.7(3)	O6^{12}	O6	K3^{12}	11(10)
O3	K1	O3^{7}	71.96(19)	O6^{12}	O6	K3	164(10)

^{1}2-x,+y,-z; ^{2}5/2-x,1/2-y,-z; ^{3}1+x,+y,+z; ^{4}3/2-x,1/2-y,1-z; ^{5}1/2-x,1/2-y,1-z; ^{6}-1+x,+y,+z; ^{7}1/2+x,1/2-y,+z; ^{8}-1/2+x,1/2-y,+z; ^{9}1-x,+y,1-z; ^{10}+x,1-y,+z; ^{11}1-x,1-y,1-z; ^{12}-x,1-y,1-z; ^{13}-1+x,1-y,+z; ^{14}-1/2+x,1/2+y,+z

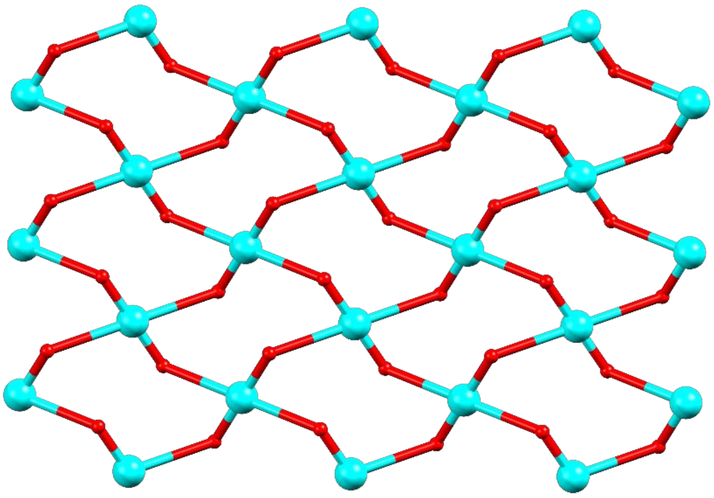

Figure S10. Topological representation of simplified 2D net in **CP1**, showing the 4-connected {Ag_2O} nodes (cyan) linked by msba ligands (red) to generate a square lattice (sql) topology.

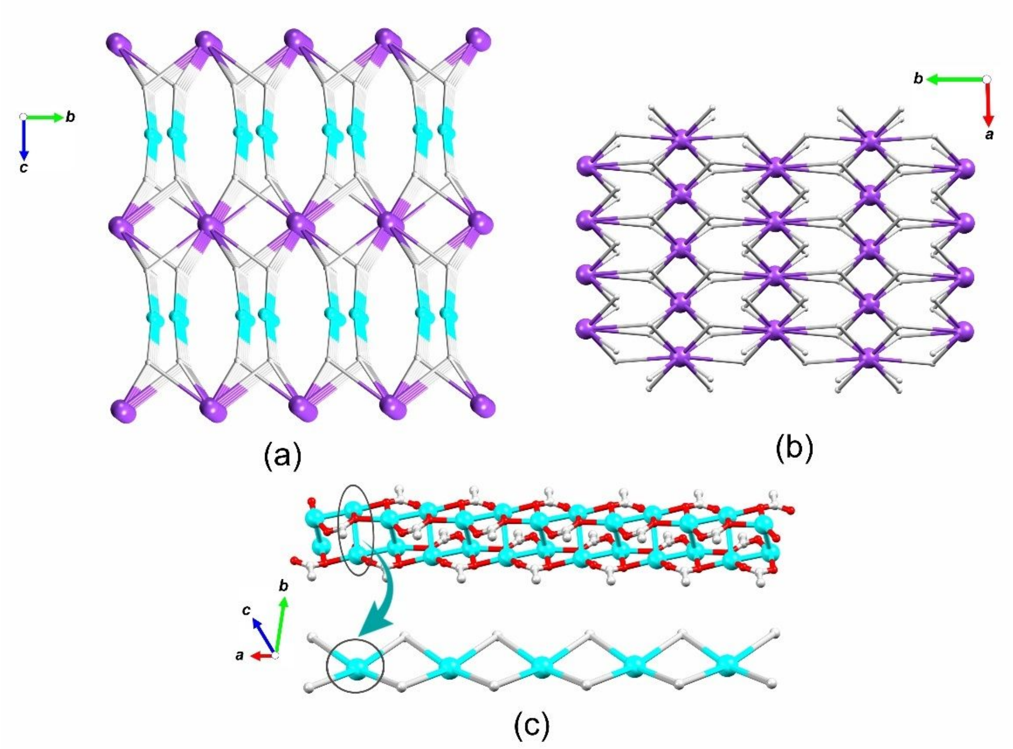

Figure S11. Topological representation of simplified 3D net in **CP2**. (a) Simplified topological model highlighting the role of potassium ions (purple) in defining the 3D metal-organic framework. (b) Simplified topological model (potassium layers) showing the uninodal 6-connected net with a hexagonal lattice (hxl) topology. (c) Simplified topological model showing {Ag_2O} clusters (cyan) interconnecting the 2D layers.

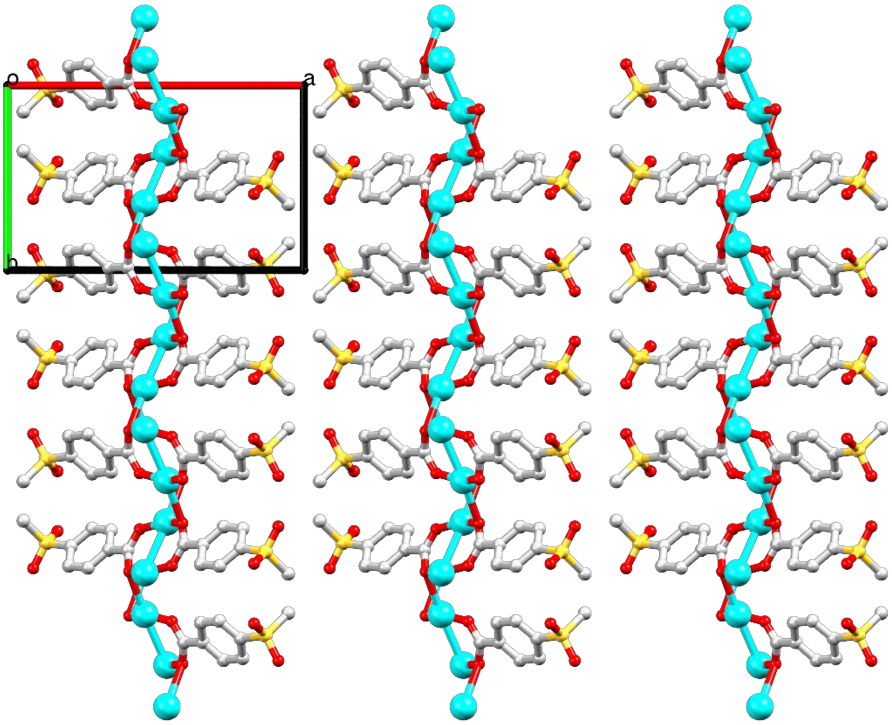

Figure S12. Representation of layers in **CP1** stacked along the crystallographic *a* axis, showing a packed lamellar structure with negligible solvent-accessible voids. Ag cyan balls, C gray, O red, S yellow.

Table S6. Electron Density Corresponding to Disordered Solvent Molecules Masked in the Unit Cell of **CP2**.

Number	X	Y	Z	Volume	Electron count	Content
1	-0.733	0.000	0.000	188.7	129.8	12 H_2O
2	-0.743	0.500	0.000	188.7	129.8	12 H_2O

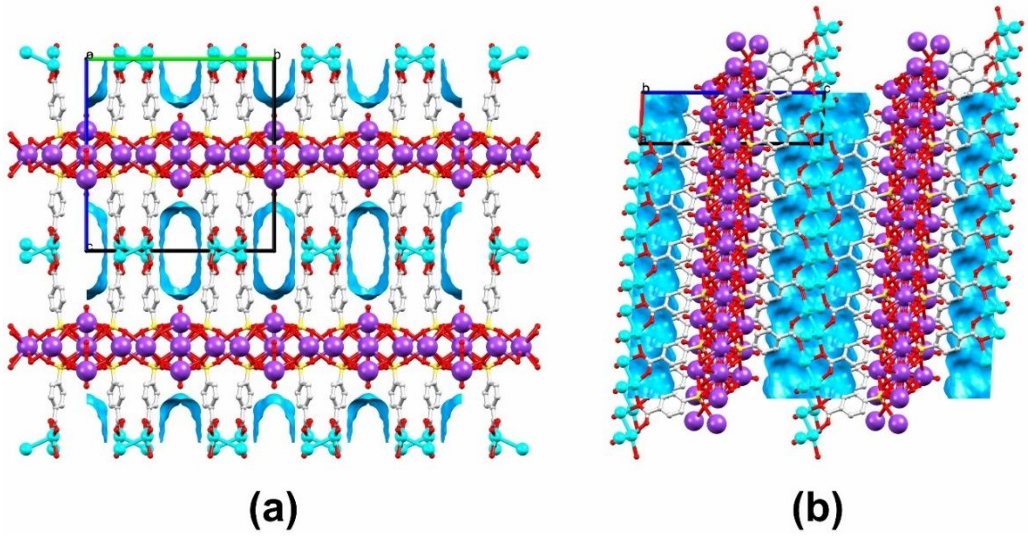

Figure S13. Crystal packing representation of **CP2** highlighting solvent-accessible voids. (a) View along the *a* axis, showing the packing relative to the *bc* plane. (b) View along the *b* axis, showing the packing relative to the *ac* plane. Ag cyan balls, K purple, O red, C gray, S yellow.

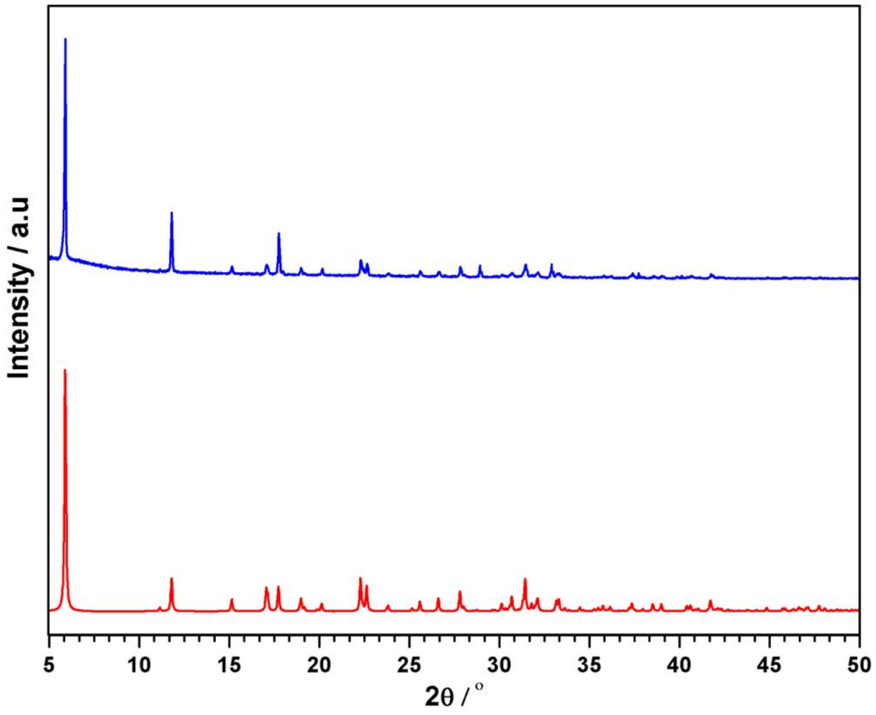

Figure S14. Powder X-ray diffraction patterns of **CP1** (blue - experimental, red - simulated).

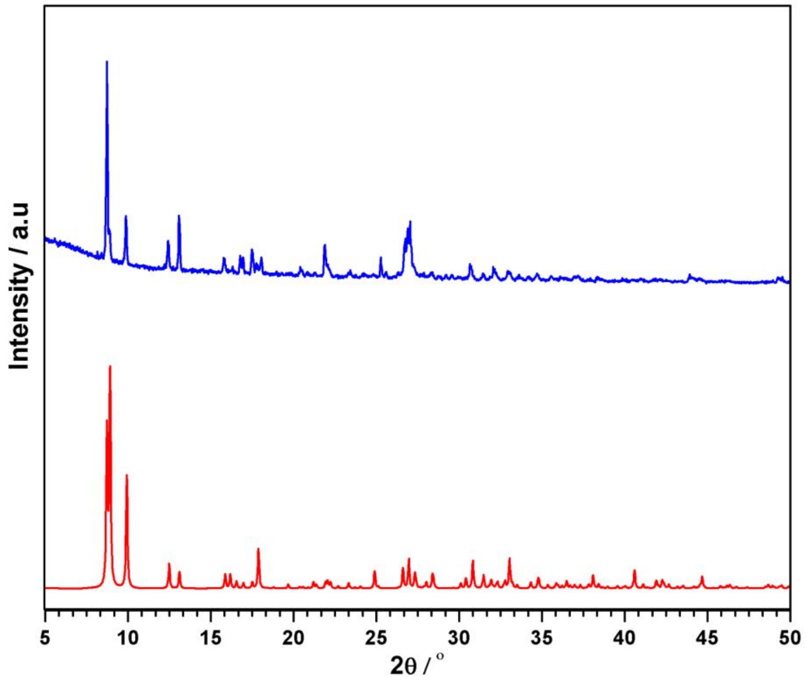

Figure S15. Powder X-ray diffraction patterns of **CP2** (blue - experimental, red - simulated considering a preferred orientation in the (020) plane).

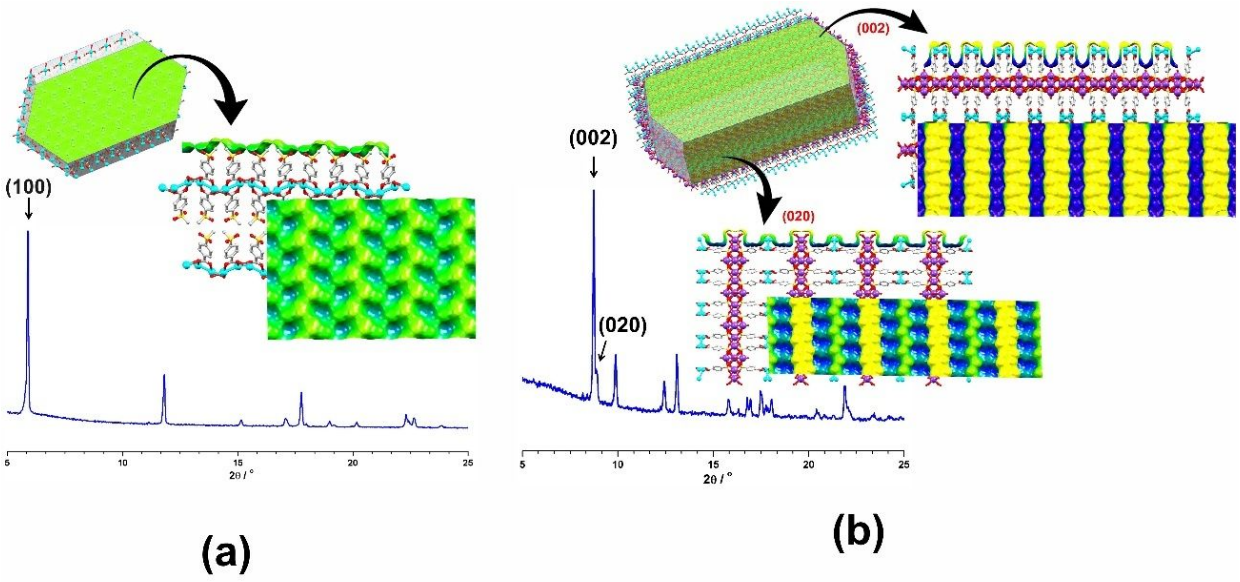

Figure S16. Experimental X-ray diffraction patterns of **CP1** (a) and **CP2** (b), highlighting their predominant exposed surfaces in crystallites. (a) Predominant exposure of the hydrophobic (100) crystallographic surface (indicated in green) in **CP1**. (b) Predominant exposure of the hydrophilic (002) and (020) crystallographic surfaces (indicated in multiple colors) in **CP2**.

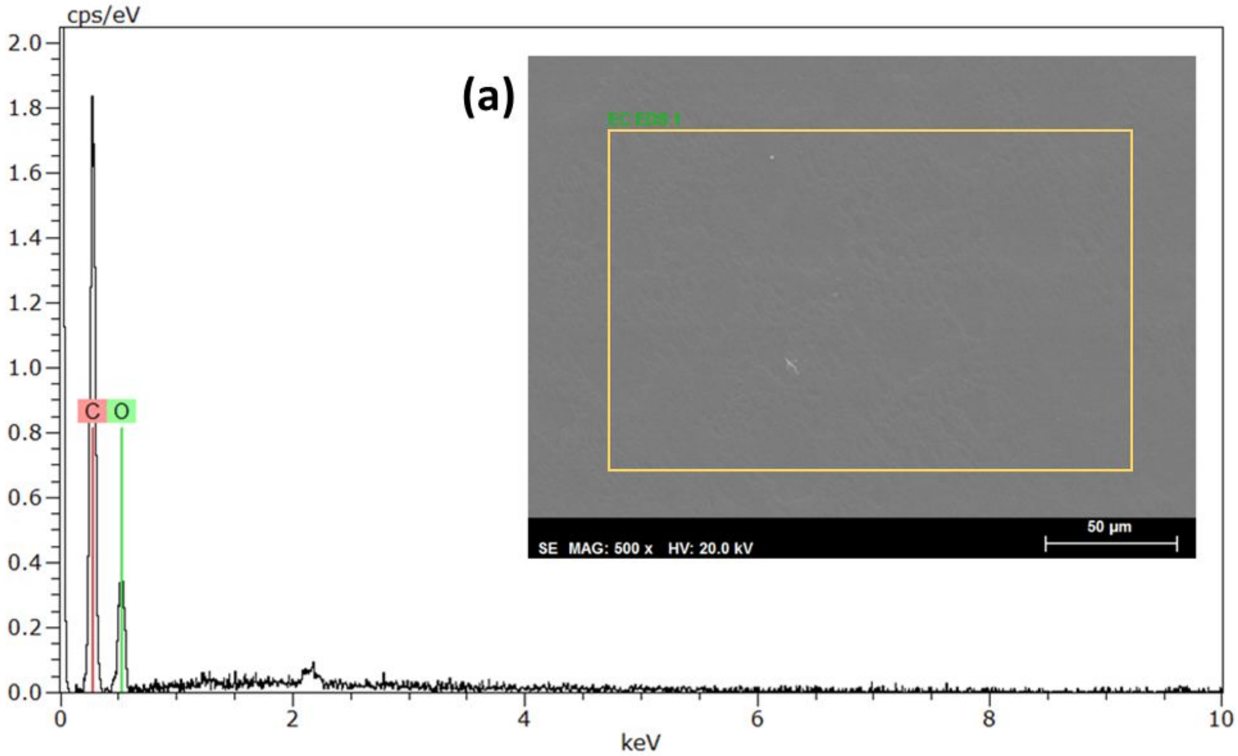

Figure S17. SEM image at 500× magnification of $[EC]_n$ biopolymer film with (a) EDS analysis of elemental composition.

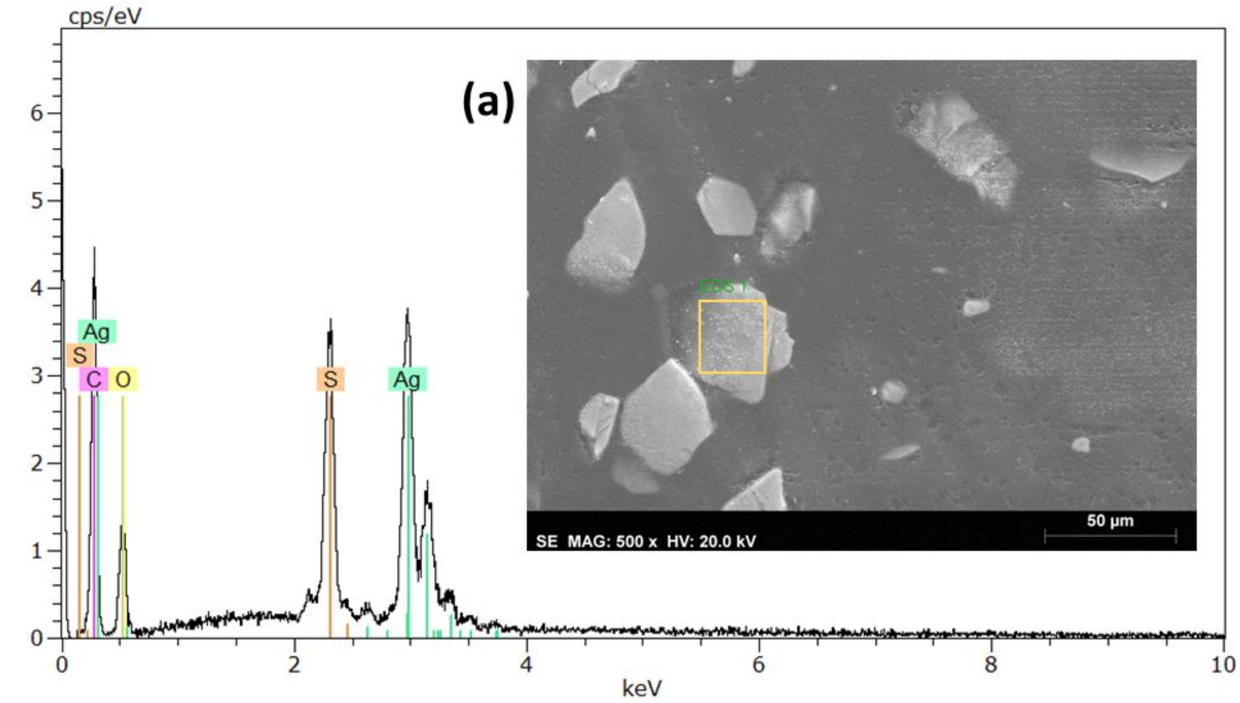

Figure S18. (a) SEM image at 500× magnification of **CP1**$^{(5\%)}$@$[EC]_n$ biopolymer film with (b) EDS analysis of elemental composition.

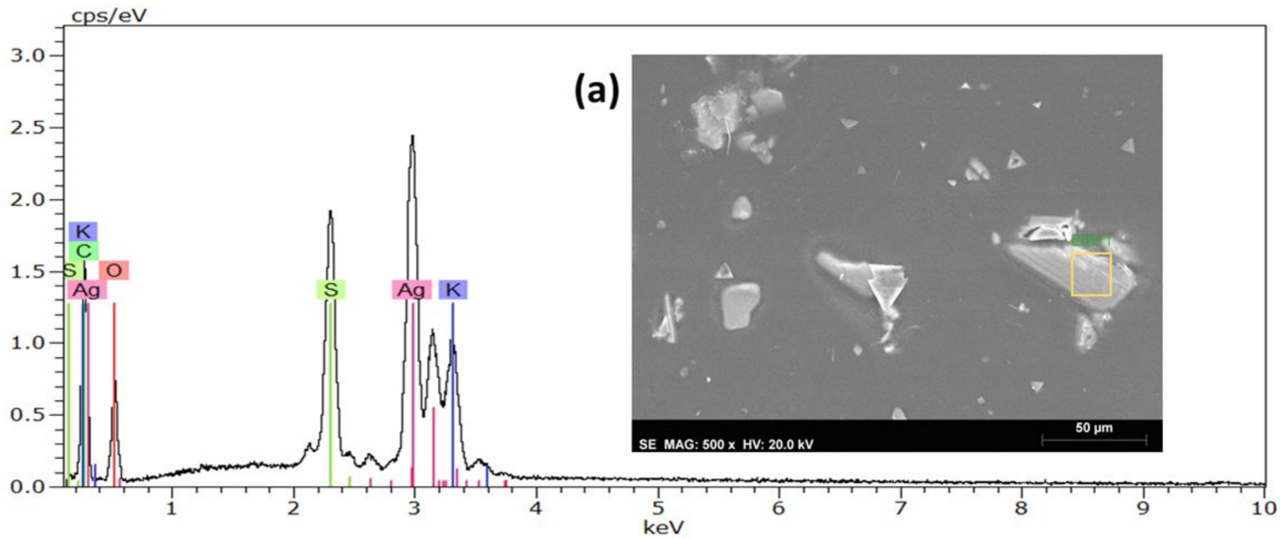

Figure S19. (a) SEM image at 500× magnification of **CP2**$^{(5\%)}$@[EC]$_n$ biopolymer film with a spectrum showing EDS analysis of elemental composition.

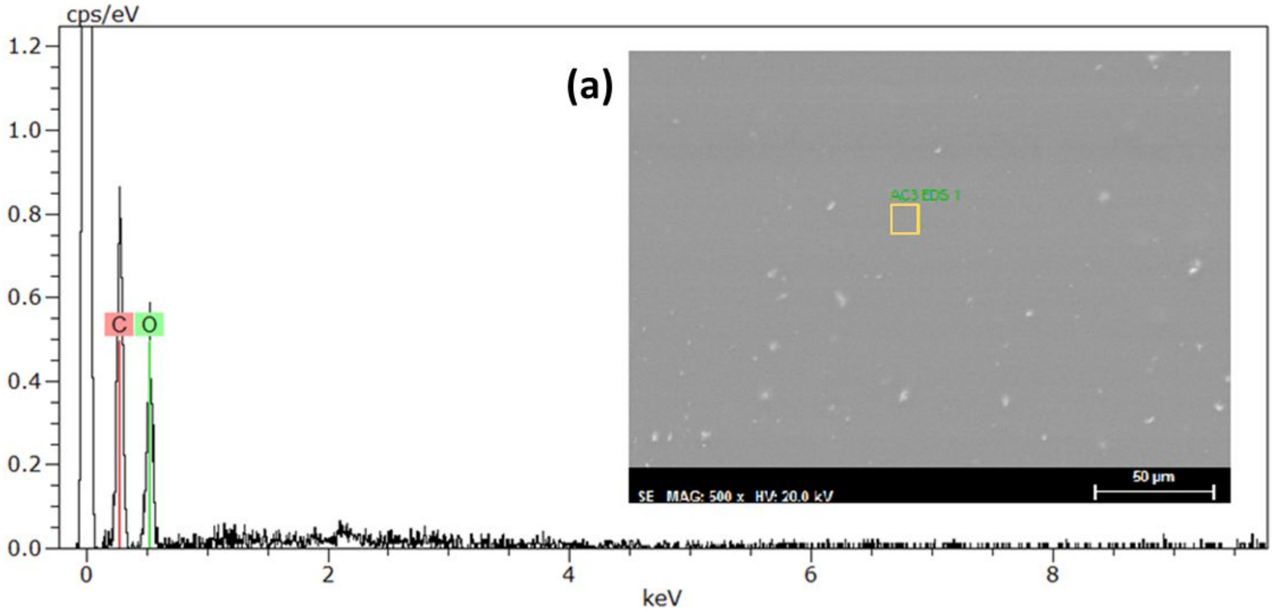

Figure S20. (a) SEM image at 500× magnification of [CA]$_n$ biopolymer film with a spectrum showing EDS analysis of elemental composition.

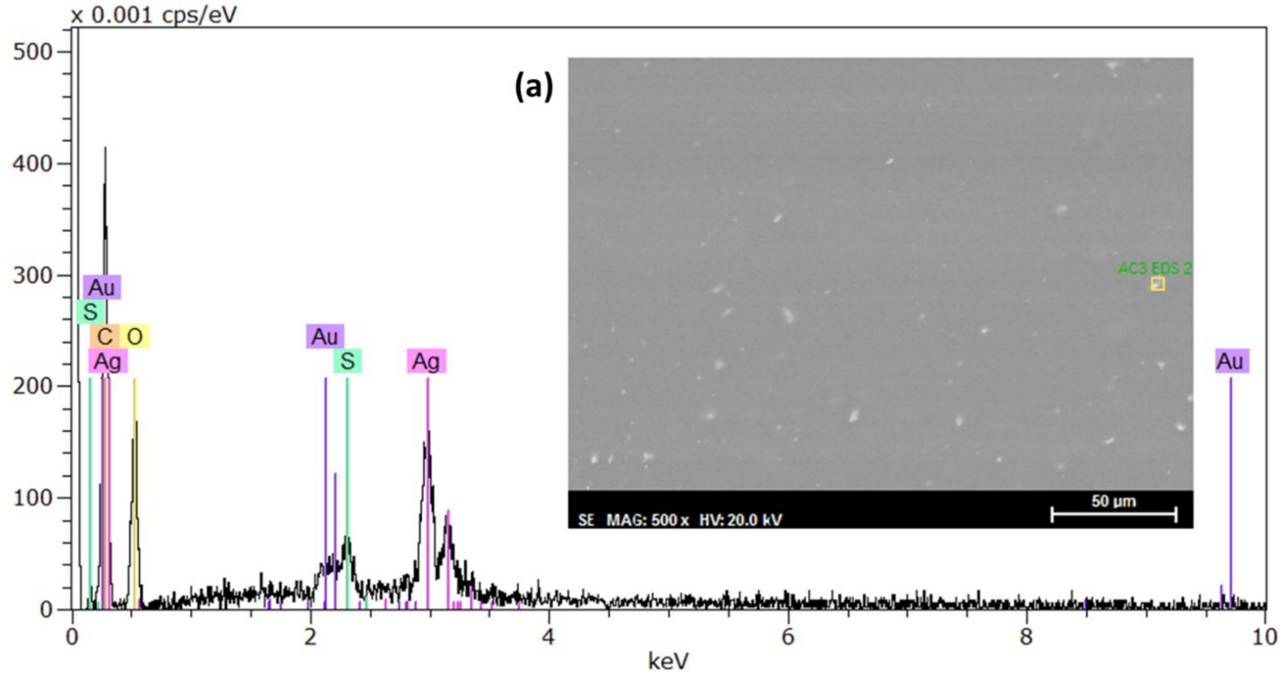

Figure S21. (a) SEM image at 500× magnification of **CP1**$^{(5\%)}$@$[CA]_n$ biopolymer film with a spectrum showing EDS analysis of elemental composition.

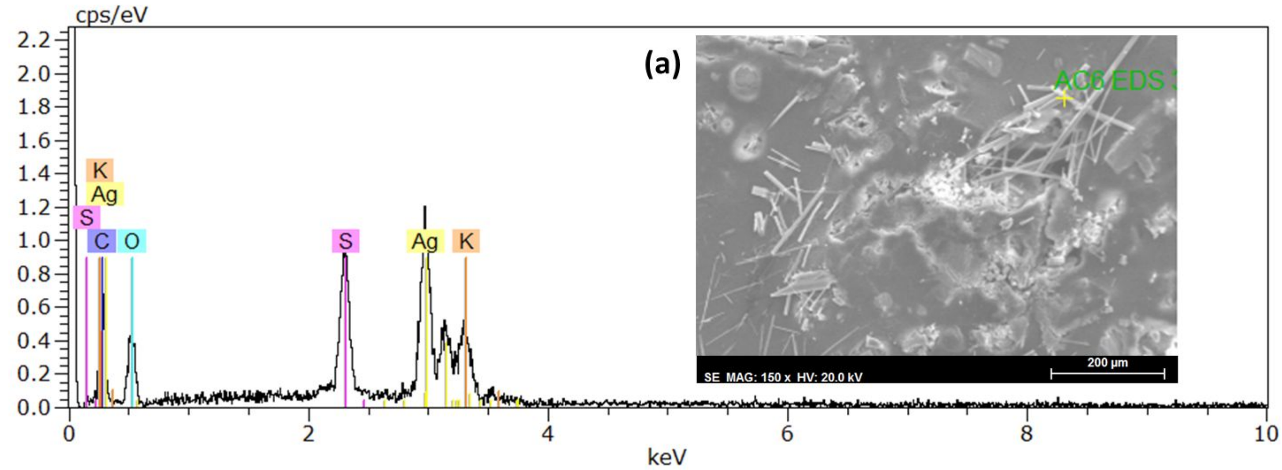

Figure S22. (a) SEM image at 500× magnification of **CP2**$^{(5\%)}$@$[CA]_n$ biopolymer film with a spectrum showing EDS analysis of elemental composition.

Mechanical Analysis

Tensile strength (TS) and percentage of elongation at break (EB) were evaluated using an Instron 5966 equipment (Instron, Norwood, Massachusetts, EUA) with a load cell of 1 ton based on the ASTM D-882-10 standard method (2010), using film samples with dimensions of approximately 80 mm × 10 mm × 0.04 mm. Before testing, strip thickness was measured at 5 points (in the film area). Force and distance were recorded during the extension at 10 mm min^{-1} up to the breaking point.

The TS and EB values of the films were calculated by eqs. (*1*) and (*2*), respectively.

$$TS = \frac{F}{W \times T} \qquad (eq.\ 1)$$

where, *F* is the maximum TS of the film (N), and *W* and *T* are the width and thickness of the film, respectively.

$$EB = \frac{L_1}{L_0} \times 100\% \qquad (eq.\ 2)$$

where, L_1 is the length of the film after stretching, L_0 is the original length of the film.

Table S7. Mechanical Properties of $[CA]_n$ Films Obtained with Different Plasticizers.

Sample	Tensile Strength	Elongation at break (mm)	Young's Modulus (MPa)
$[CA]_n$	31.50	1.62	2181.01
$[CA]_n$ + 1% Glycerol	31.90	2.11	1733.23
$[CA]_n$ + 5% Glycerol	27.02	1.45	1718.68
$[CA]_n$ + 10% Glycerol	31.60	1.79	2135.66
$[CA]_n$ + 1% PEG	34.78	2.17	1918.58
$[CA]_n$ + 5% PEG	26.32	1.71	1968.85
$[CA]_n$ + 10% PEG	26.05	1.45	2012.73
$[CA]_n$ + 1% Sorbitol	18.94	1.77	1124.05
$[CA]_n$ + 5% Sorbitol	6.04	1.36	627.75
$[CA]_n$ + 10% Sorbitol	0.62	0.28	224.42

Table S8. Mechanical Properties of $[EC]_n$ Films Obtained with Different Plasticizers.

Sample	Tensile Strength	Elongation at break (mm)	Young's Modulus (MPa)
$[EC]_n$	23.73	0.94	2464.33
$[EC]_n$ + 1% Glycerol	18.20	0.90	2219.50
$[EC]_n$ + 5% Glycerol	23.83	1.89	1382.33
$[EC]_n$ + 10% Glycerol	22.60	1.42	1757.75
$[EC]_n$ + 1% PEG	31.75	1.85	1799.75
$[EC]_n$ + 5% PEG	18.23	0.92	2103.25
$[EC]_n$ + 10% PEG	14.96	0.94	1595.75
$[EC]_n$ + 1% Sorbitol	28.66	1.95	1876.25
$[EC]_n$ + 5% Sorbitol	21.53	1.37	1788.00
$[EC]_n$ + 10% Sorbitol	21.90	1.49	1623.25

Rheological Analysis

Viscosity measurements were performed on an Anton Paar MCR 92 rheometer in continuous flow mode at 25 °C, using a shear rate range from 0.01 to 1000 s^{-1} and a 50 mm cone-plate geometry. Reliable measurements were obtained only for the $[EC]_n$-based mixtures, since solvent evaporation prevented consistent analysis of the $[CA]_n$ systems. The $[EC]_n$ mixtures exhibited typical shear-thinning (pseudoplastic) behavior, while the incorporation of the silver dopant resulted in lower viscosity values, likely due to interference with intermolecular polymer interactions.

Additional Antimicrobial Data

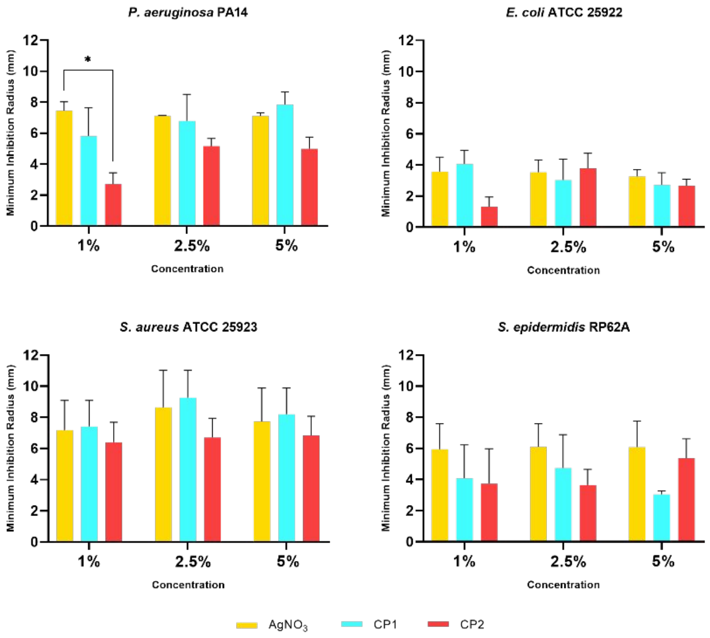

Figure S23. Not normalized antibacterial activity of $[CA]_n$ films doped with 1, 2.5, and 5% of $AgNO_3$ (positive control, **CP1**, and **CP2**, against four bacterial pathogens, namely the Gram-positive *P. aeruginosa* PA14 and *E. coli* ATCC 25922 and the Gram-negative *S. aureus* ATCC 25923 and *S. epidermidis* RP62A. Results are depicted as the minimum radius of the growth inhibition halos with error bars representing the SEM. Significant statistical differences: P < 0.05 (*).

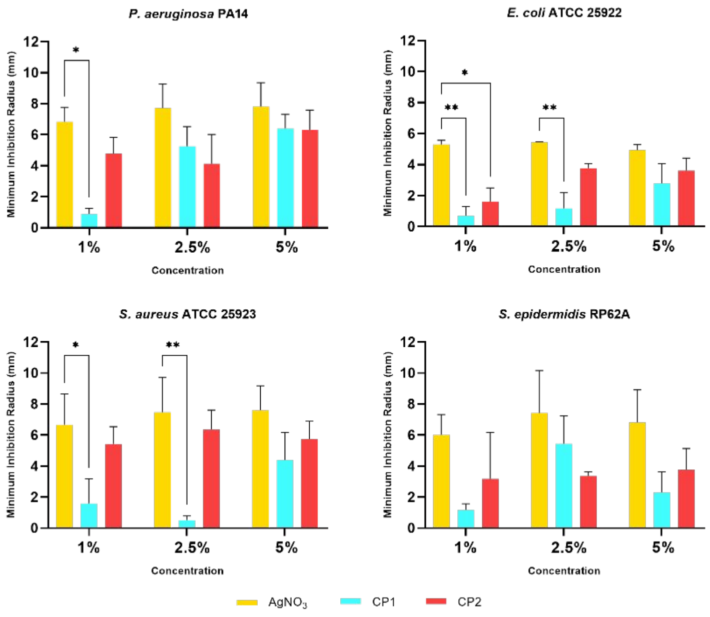

Figure S24. Not normalized antibacterial activity of $[EC]_n$ films doped with 1, 2.5, and 5% of $AgNO_3$ (positive control), **CP1**, and **CP2**, against four bacterial pathogens, namely the Gram-positive *P. aeruginosa* PA14 and *E. coli* ATCC 25922 and the Gram-negative *S. aureus* ATCC 25923 and *S. epidermidis* RP62A. Results are depicted as the minimum radius of the growth inhibition halos with error bars representing the SEM. Significant statistical differences: P < 0.05 (*), P < 0.01 (**).

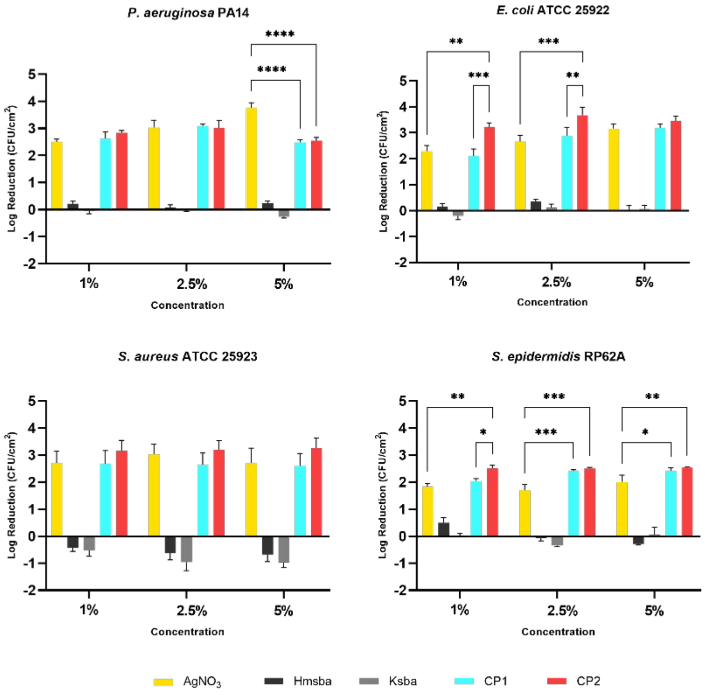

Figure S25. Not normalized biofilm inhibition activity of [CA]n films doped with 1, 2.5, and 5% of $AgNO_3$ (positive control), Hmsba (organic ligand control for **CP1**), KHsba (organic ligand control for **CP2**), **CP1**, and **CP2**, against four bacterial pathogens, namely the Gram-positive *P. aeruginosa* PA14 and *E. coli* ATCC 25922 and the Gram-negative *S. aureus* ATCC 25923 and *S. epidermidis* RP62A. Results are depicted as log reductions (relative to the non-doped matrix control) with error bars representing the SEM. Significant statistical differences: $P < 0.05$ (*), $P < 0.01$ (**), $P < 0.0001$ (****).

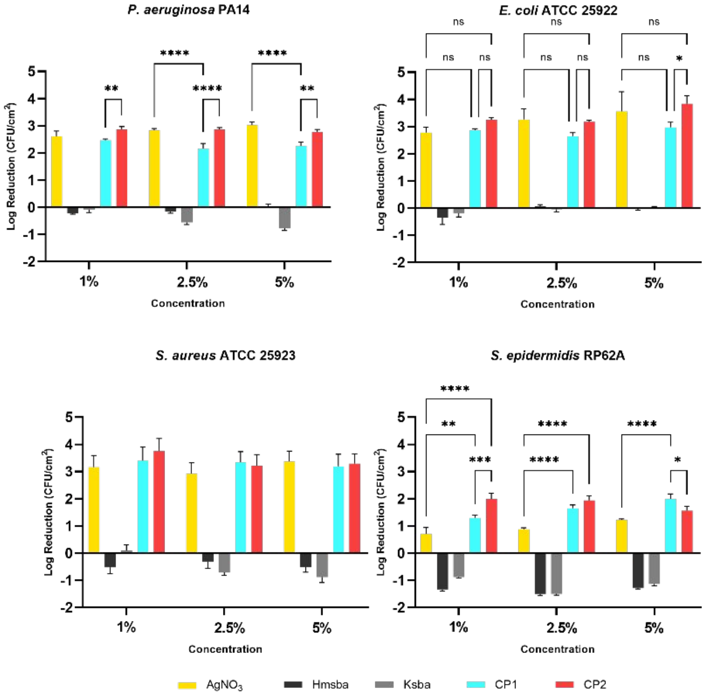

Figure S26. Not normalized biofilm inhibition activity of [EC]n films doped with 1, 2.5, and 5% of $AgNO_3$ (positive control), Hmsba (organic ligand control for **CP1**), Ksba (organic ligand control for **CP2**), **CP1**, and

CP2, against four bacterial pathogens, namely the Gram-positive *P. aeruginosa* PA14 and *E. coli* ATCC 25922 and the Gram-negative *S. aureus* ATCC 25923 and *S. epidermidis* RP62A. Results are depicted as log reductions (relative to the non-doped matrix control) with error bars representing the SEM. Significant statistical differences: P < 0.05 (*), P < 0.01 (**), P < 0.0001 (****).

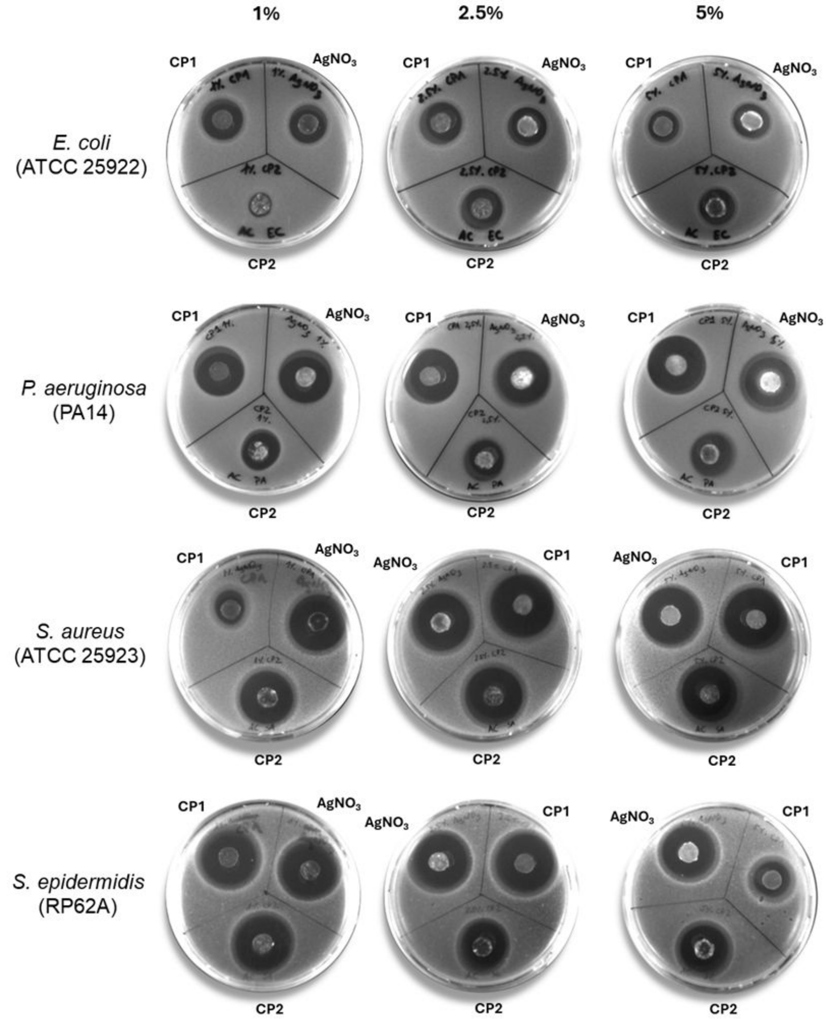

Figure S27. Examples of inhibition halos obtained for *E. coli* ATCC 25922, *P. aeruginosa* PA14, *S. aureus* ATCC 25923 and *S. epidermidis* RP62A with $[CA]_n$ films doped with $AgNO_3$ and compounds **CP1** and **CP2** (1, 2 and 5%).

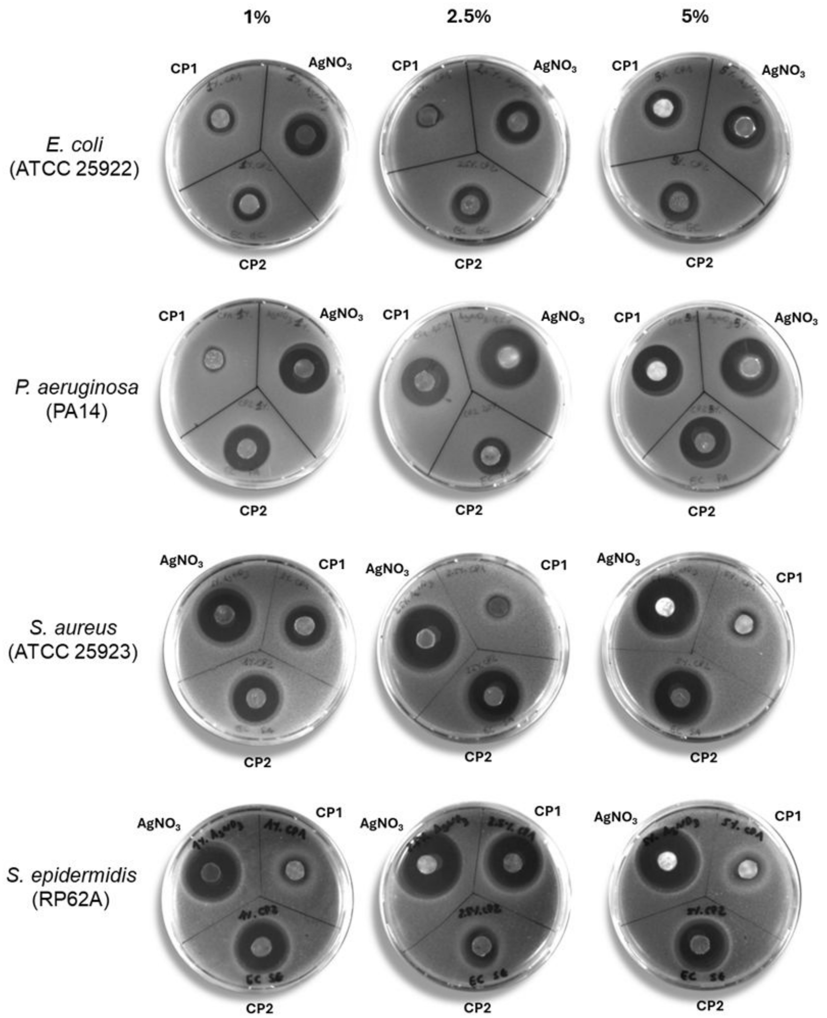

Figure S28. Examples of inhibition halos obtained for *E. coli* ATCC 25922, *P. aeruginosa* PA14, *S. aureus* ATCC 25923 and *S. epidermidis* RP62A with $[EC]_n$ films doped with $AgNO_3$ and compounds **CP1** and **CP2** (1, 2 and 5%).

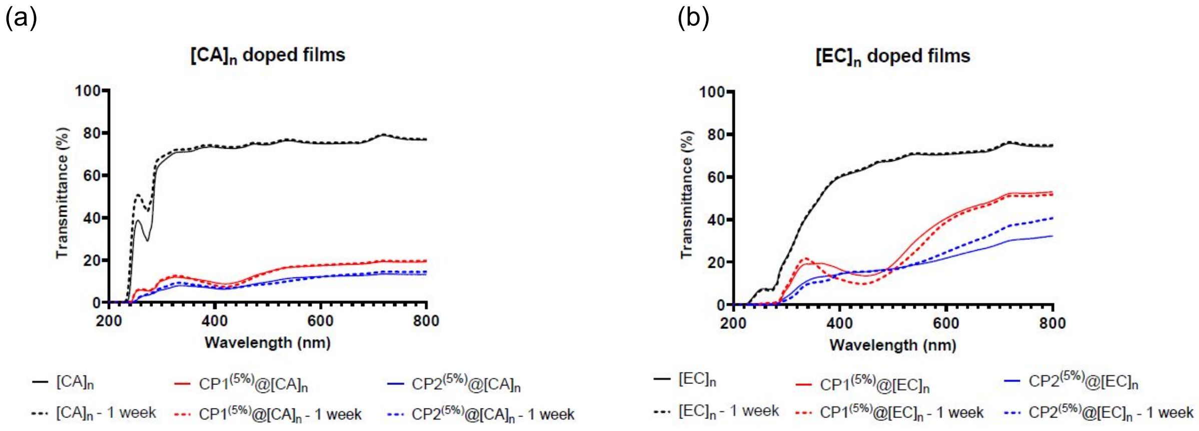

Figure S29. UV-vis transmittance spectra of $[CA]_n$ (a) and $[EC]_n$ (b) controls, as well as hybrid **CP1**$^{(5\%)}$@$[EC]_n$ and **CP2**$^{(5\%)}$@$[EC]_n$ (a), **CP1**$^{(5\%)}$@$[CA]_n$ and **CP2**$^{(5\%)}$@$[CA]_n$ (b) biopolymer films before and after 1-week exposure to sunlight.

$[CA]_n$

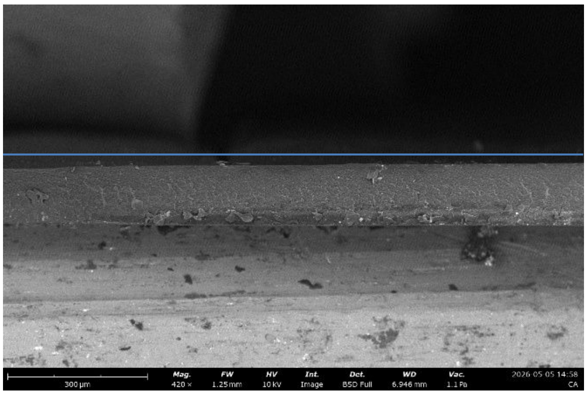

$[EC]_n$

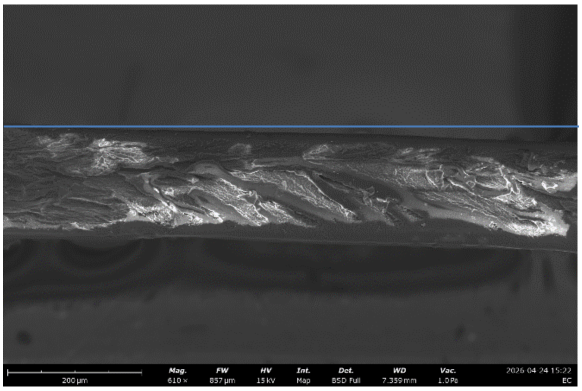

$CP1^{(5\%)}@[CA]_n$

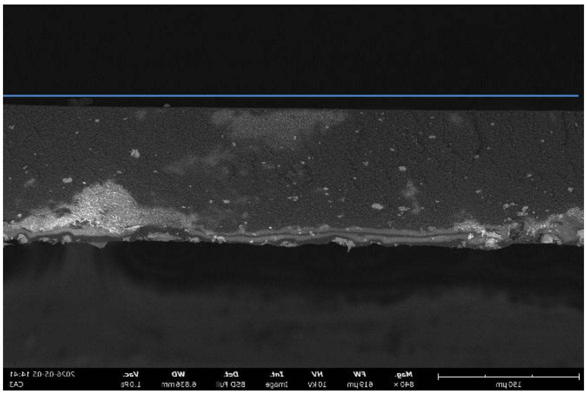

$CP1^{(5\%)}@[EC]_n$

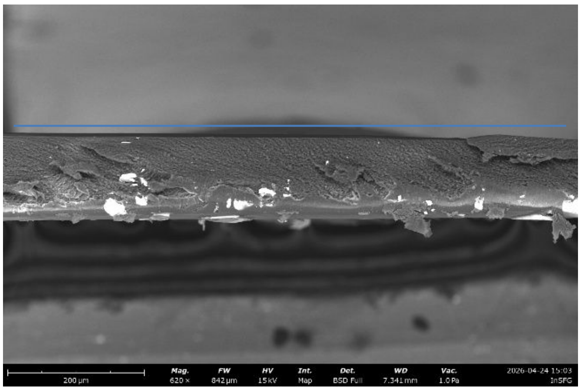

$CP2^{(5\%)}@[CA]_n$

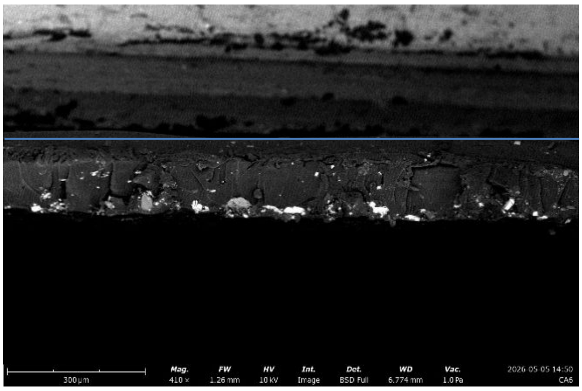

$CP2^{(5\%)}@[EC]_n$

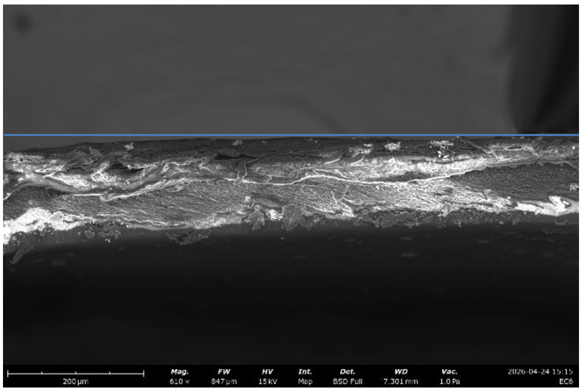

Figure S30. SEM images of the film's cross-section of different biopolymer films, showing the formation of agglomerates.
